# Host Adaptation and Evolutionary Analysis of *Zaire ebolavirus*: Insights From Codon Usage Based Investigations

**DOI:** 10.3389/fmicb.2020.570131

**Published:** 2020-11-05

**Authors:** Wen Luo, Ayan Roy, Fucheng Guo, David M. Irwin, Xuejuan Shen, Junbin Pan, Yongyi Shen

**Affiliations:** ^1^Center for Emerging and Zoonotic Diseases, College of Veterinary Medicine, South China Agricultural University, Guangzhou, China; ^2^Guangdong Laboratory for Lingnan Modern Agriculture, Guangzhou, China; ^3^Department of Biotechnology, Lovely Professional University, Phagwara, India; ^4^Department of Laboratory Medicine and Pathobiology, University of Toronto, Toronto, ON, Canada; ^5^Banting and Best Diabetes Centre, University of Toronto, Toronto, ON, Canada; ^6^Key Laboratory of Zoonosis Prevention and Control of Guangdong Province, Guangzhou, China

**Keywords:** codon usage, ebolavirus, host reservoir, codon adaptation index, genetic diversity

## Abstract

Ebola virus (EBOV) has caused several outbreaks as the consequence of spillover events from zoonotic sources and has resulted in huge death tolls. In spite of considerable progress, a thorough know-how regarding EBOV adaptation in various host species and detailed information about the potential reservoirs of EBOV still remains obscure. The present study was executed to examine the patterns of codon usage and its associated influence in the adaptation of EBOV to potential hosts that dwell in Africa, the origin of the viral outbreaks. Correspondence analysis (CA) revealed that the codon usage signature in EBOV is a complex interplay of factors including compositional bias and natural selection, with the latter having a more pronounced impact. Low codon usage bias in EBOV indicates a flexibility of the viruses in adapting to diverse range of hosts with different codon usage architectures. EBOV adaptation in potential hosts, as estimated by codon adaptation index (CAI) and relative codon deoptimization index (RCDI), revealed that the viruses were relatively better adapted to African primates than other mammals examined, which might account for the high fatality rate of primates owing to EBOV infection. Bats have been speculated as natural reservoirs of EBOV. In the present analysis it was interesting to note that EBOV displayed lower degrees of adaptation, as estimated by CAI and RCDI, with bats in comparison to the primate hosts. Lower degrees of adaptation might contribute to long-term co-existence and circulation of the viral pathogens in bat populations. Codon usage patterns of EBOV isolates associated with different outbreaks varied significantly, with discrete patterns between the West and Central African isolates. Additional evolutionary analyses indicated that the West African Epidemic began with an initial spillover infection and there was more than one population of EBOV circulating in the natural reservoir in the Democratic Republic of the Congo. The present study yields valuable information regarding the possible circulation of EBOV in various African mammals.

## Introduction

Ebolaviruses are non-segmented, negative-sense, single-stranded RNA viruses belonging to family *Filoviridae* in the order *Mononegavirales*. The genus *Ebolavirus* contains six species, namely, *Bombali ebolavirus*, *Bundibugyo ebolavirus*, *Sudan ebolavirus*, *Taï Forest ebolavirus*, *Reston ebolavirus*, and *Zaire ebolavirus* (Burk et al., 2016; Goldstein et al., 2018). The 19 kb (approximately) viral genome encodes seven essential proteins, which are nucleoprotein (NP), glycoprotein (GP), L-polymerase (L) protein, and the viral proteins (VP) VP24, VP30, VP35, and VP40 (Baseler et al., 2017). Since 1976, Ebolaviruses have caused more than 20 outbreaks, with the majority have caused by Ebola virus (EBOV), the member of the species *Z. ebolavirus* (CDC, 2019). EBOV has been associated with the recent most severe outbreak that occurred in West Africa between 2013 and 2016, which infected more than 28,000 humans and had a death toll of 11,325 (CDC, 2019). The most recent EBOV outbreak is currently ongoing in the Democratic Republic of the Congo and is also caused by EBOV (CDC, 2019).

Despite greater than 40 years of continued outbreaks, a thorough understanding of EBOV origin, epidemicity and host adaptation still remains obscure (Olival and Hayman, 2014). Bats have long been speculated as being the reservoirs for EBOV (Leroy et al., 2005; Goldstein et al., 2018). Although viral replication can be induced in bats through experimental inoculation (Swanepoel et al., 1996), live EBOV isolates have never been reported in any wild bat species to date (Caron et al., 2018). Thus, proper identification of the reservoir hosts of EBOV and apt detection of the possible routes of viral spillover to human population are demanded at deeper levels. The African forest ecosystem exhibits a large level of biodiversity, suggesting that apart from bats other mammals might play crucial roles in the maintenance and circulation of EBOV and facilitate the spillover events to humans (Caron et al., 2018). A detailed investigation regarding EBOV adaptation in these species promises to confer valuable insights into the transmission and epidemiology of the virus.

Viruses, owing to their small sized genomes, largely depend on the cellular machinery and metabolism of their hosts for efficient replication, protein synthesis and assembly, and thus their fitness is associated with their hosts’ cellular environment (Kumar N. et al., 2016). Since the genetic code is degenerate, preferential use of synonymous codons (codons encoding the same amino acid) leads to a codon usage bias in genes and genomes (Plotkin and Kudla, 2011). Bias in codon usage is evident in all forms of life encompassing not only prokaryotic and eukaryotic organisms but also viruses (Plotkin and Kudla, 2011). Codon usage signatures in viral genomes have been reported to be shaped by multiple determinants, with major impacts from mutational pressure and translational selection constraints exerted by the hosts that harbor the viruses (Butt et al., 2016). Viruses coevolve and mimic host codon usage patterns to efficiently utilize host resources and adapt to proficiently use the host’s tRNA. Host adaptation is an extremely important factor that influences the fitness and survival of viral pathogens (Butt et al., 2016). High competence of a virus inside a particular host increases the magnitude of the infection caused by it (Butt et al., 2016).

Considerable progress has been made in the areas of molecular evolution, host adaptation, transmission dynamics and pathogenesis of EBOV (Holmes et al., 2016; McMullan et al., 2019). However, we still need to better understand the facets of viral adaptation before we can estimate the potential for cross-species jumps that might lead to future outbreaks (Caron et al., 2018). Accordingly, our present research was undertaken to investigate the codon usage patterns of EBOV, identify any correlations with potential hosts, and simultaneously explore the possibilities for circulation and adaptation of these viruses across various susceptible reservoir host species. Genetic diversity plays a key role in shaping the evolutionary dynamics of viral genomes (Banerjee et al., 2012). Genetic alterations contribute to the ability of a viral pathogen to efficiently respond to changes in the host environment and its ability to adapt to the host selection pressure (Banerjee et al., 2012). The present study, aimed to explore the genetic diversity among EBOV promises to untangle their evolutionary patterns and facilitate predictive analysis associated with the control of imminent Ebola outbreaks.

## Materials and Methods

### Data Collection

A total of 308 complete EBOV genome sequences were obtained from NCBI GenBank^1^ (detailed in Supplementary Table 1). For each genome of EBOV, ORFs were concatenated (NP + VP35 + VP40 + GP + VP30 + VP24 + L) (Cristina et al., 2015) and then aligned using MAFFT v7.245 software (Katoh and Toh, 2010).

Apart from humans, Ebolaviruses have mostly been isolated from Gorilla (*Gorilla gorilla gorilla*) and Chimpanzee (*Pan troglodytes*) (Leroy et al., 2004; Bermejo et al., 2006). Since other mammals residing in Africa might harbor EBOV, and act as potential hosts for the virus, we also considered them (mammals with complete genome sequences available publicly) in the present analysis. In order to complement for the mammals (residing in Africa) with unavailable genome sequences, we further included closely related species (with complete genome sequences available publicly) from other continents belonging to the orders Chiroptera, Rodentia and Primates. A detailed analysis of EBOV adaptation in these species promises to provide valuable insights on EBOV adaptation in closely related species that reside in Africa (with unavailable complete genomes). Annotated coding sequences from the complete genomes of all of the concerned organisms were obtained from the RefSeq database^2^ (detailed in Supplementary Table 1).

### Computation of the Base Compositional Features

The coding sequences of the EBOV genomes and associated potential reservoir species were used for the analysis of base compositional features. The overall frequencies of nucleotides (A, U, C, and G%), frequencies of nucleotides at the third positions of synonymous codons (A3s, C3s, U3s, and G3s%), base composition of G and C at first (GC1), second (GC2), and third (GC3) positions of codons and overall GC content were calculated using the CodonW program^3^. Relative synonymous codon usage (RSCU), an index of heterogeneous usage of synonymous codons, refers to the ratio of the observed frequency of a particular codon to its expected frequency in the case of uniform synonymous codon usage (dos Reis et al., 2003).

RSCU is computed as:

$$RSCU=\frac{\mathrm{Frequency}\mathrm{of}\mathrm{codon}}{\mathrm{Expected}\mathrm{frequency}\mathrm{of}\mathrm{codon}}$$

(ifcodonusagewasuniform)

RSCU values of the EBOV coding sequences were calculated using CodonW. Codons with RSCU > 1.6 were considered as over-represented whereas, codons with RSCU < 0.6 reflected under-represented ones (Wong et al., 2010).

### Estimation of Effective Number of Codons

Effective number of codons (ENC), an estimate of codon usage in genes and genomes, is expressed as:

$$\mathrm{ENC}=2+\frac{9}{{\text{F}}_{2}}+\frac{1}{{\text{F}}_{3}}+\frac{5}{{\text{F}}_{4}}+\frac{3}{{\text{F}}_{6}}$$

where, Fk (*k* = 2, 3, 4 or 6) refers to the average value of Fk pertaining to *k*-fold degenerate amino acids and F signifies the probability of two randomly selected codons for an amino acid being identical. ENC ranges from 20 (a case of extreme codon usage bias when an amino acid is coded by a single codon) to 61 (an instance depicting absence of codon bias when an amino acid is encoded by all its synonymous codons) (Wright, 1990). CodonW was employed to calculate the ENC values of the viral genes. GC3 versus ENC plots have been suggested to be instrumental in studying codon usage variations among genes and genomes (Wright, 1990). GC3-ENC plot for the EBOV coding sequences was generated using the R software package^4^.

### Neutrality Plot

Neutrality plot analysis, a measure of neutral evolution, was executed to explore the magnitude of genomic compositional constraint and natural selection operating on the EBOV coding sequences (Sueoka, 1988). GC3 values (*x*-axis) of the viral genes were plotted against the respective GC12 values (*y*-axis) to generate the neutrality plots. It has been suggested that the slope of the plot reflects the degree of compositional constraint operating on the genes of interest (Sueoka, 1988). The neutrality plot for the EBOV coding sequences was generated using the R software package^5^.

### Computation of Translational Selection Index (P2)

Translational selection index (P2) reflects the extent of interaction between a codon and its respective anticodon and is frequently used to determine the degrees of translational selection acting on genes of interest (Gatherer and McEwan, 1997). P2 for the EBOV coding sequences was calculated as:

$$P2=\frac{WWC+\mathrm{SSU}}{\text{WWY}+\mathrm{SSY}}$$

where, W denotes the frequency of Adenine [A] or Uracil [U], S signifies the frequency of Cytosine [C] or Guanine [G], and Y reflects the frequency of Cytosine [C] or Uracil [U].

### Codon Adaptation Index

Codon adaptation index (CAI), an efficient index of probable gene expression levels, portrays the degrees of viral adaptation to the host cellular niche (Puigbo et al., 2008). CAI values of the concerned EBOV coding sequences were estimated employing a standalone version of CAIcal server^6^, with respect to the codon usage patterns of the potential associated hosts (Puigbo et al., 2008). CAI values range between 0 and 1 with higher CAI values signifying better viral adaptation with the host cellular machinery (Puigbo et al., 2008). Kruskal Wallis test was employed to assess the statistical significance of the differences between CAI values of EBOV calculated in reference to the different host species.

### Relative Codon Deoptimization Index

Relative codon deoptimization index (RCDI) is an estimate of the degree of acclimatization of a viral genome in host microcellular niche (Puigbò et al., 2010). RCDI values of the EBOV coding sequences were calculated in reference to the potential hosts using the RCDI/eRCDI server^7^ in order to determine the codon deoptimization trends by comparing the similarity of virus and host codon usage patterns. RCDI value of 1 indicates that the virus follows the host codon usage patterns and display host-adapted codon usage signatures. On the contrary, RCDI values higher than 1 signify the deoptimization of the codon usage patterns of the virus from that of its hosts (Puigbò et al., 2010). Kruskal Wallis test was used to check the statistical significance of the differences between RCDI values of EBOV calculated in reference to the different host species.

### Correspondence Analysis

Correspondence analysis is a useful multivariate statistical method employed for the identification of major sources of variation in synonymous codon usage data (Sharp and Li, 1986). In CA, every coding sequence is represented as a 59-dimensional vector with each dimension corresponding to the RSCU value of a particular codon (excluding non-synonymous AUG, UGG, and stop codons). Major trends within a dataset are explored using the measures of relative inertia and pertaining data cluster along the major axes of separation according to the variations observed. CA was performed using CodonW program.

### Correlation Analysis

Spearman’s rank correlation analyses pertaining to the RSCU data of the EBOV coding sequences (at 5% [*p* < *0.05*] and 1% [*p* < *0.01*] levels of significance) were performed employing SPSS software (version 23.0).

### Genetic Diversity and Analysis of Haplotypes in EBOV

The genetic identities of the concerned EBOV genomes were estimated using Mega v7.0 software (Kumar S. et al., 2016). Parameters including nucleotide diversity, frequency of haplotypes and haplotype diversity were estimated using DnaSP 5.10.0 software (Librado and Rozas, 2009). Median-joint networks of the EBOV ORFs were constructed with Network 5.0^8^ software.

## Results

### Base Composition Analysis of EBOV

An extensive analysis of base composition of the concerned EBOV genomes revealed that the average AU and GC contents (%) were 56.40 ± 0.06 and 43.60 ± 0.06, respectively, indicating an overall AU richness (Table 1). The observation that the mean A% (31.10 ± 0.03) and U% (25.40 ± 0.05) contents were higher than the average G% (20.30 ± 0.05) and C% (23.20 ± 0.03) contents (*p* < *0.01*) further emphasized AU bias among the EBOV genomes (Table 1). The average composition (%) of nucleotides at the third positions of synonymous codons were found to be significantly higher for U3s (37.30 ± 0.09) and A3s (39.80 ± 0.12) than C3s (25.40 ± 0.08) and G3s (23.50 ± 0.12) (*p* < *0.01*).

**TABLE 1:** Compositional features of EBOV and its potential hosts.

Genome	ENC	AU (%)	GC (%)	GC1 (%)	GC2 (%)	GC3 (%)	U (%)	C (%)	A (%)	G (%)	U3s (%)	C3s (%)	A3s (%)	G3s (%)
EBOV	55.57	56.40	43.60	51.90	40.40	38.00	25.40	23.20	31.10	20.30	37.30	25.40	39.80	23.50
*Panthera pardus* (Leopard)	54.80	47.50	52.50	56.40	42.80	56.70	21.50	26.00	26.10	26.40	28.10	37.30	25.50	34.90
*Acinonyx jubatus* (Cheetah)	55.14	48.90	51.10	55.30	41.90	54.50	22.40	25.20	26.60	25.80	29.80	35.90	26.70	33.70
*Vulpes vulpes* (Red fox)	54.75	47.90	52.10	56.30	42.70	55.80	21.70	25.90	26.20	26.20	28.90	36.70	25.80	34.30
*Rousettus aegyptiacus* (Egyptian rousette)	54.79	47.60	52.40	56.30	42.80	56.60	21.60	26.10	26.10	26.30	28.13	37.35	25.52	34.64
*Miniopterus natalensis* (Natal long-fingered bat)	54.52	48.10	51.90	56.00	42.40	56.20	21.80	25.80	26.20	26.10	28.79	36.99	25.56	34.57
*Oryctolagus cuniculus* (Rabbit)	54.15	47.00	53.00	56.50	43.20	57.90	21.20	26.40	25.80	26.60	27.10	38.00	24.90	35.50
*Otolemur garnettii* (Small-eared galago)	54.77	48.50	51.50	56.10	42.30	54.60	22.10	25.50	26.40	26.00	29.82	35.87	26.47	33.66
*Mandrillus leucophaeus* (Drill)	54.76	48.60	51.40	55.50	42.10	54.90	22.20	25.40	26.50	25.90	29.66	36.27	26.38	33.77
*Cercocebus atys* (Sooty mangabey)	54.96	48.40	51.60	56.10	42.80	54.60	21.90	25.70	26.50	25.90	29.60	35.86	26.70	33.57
*Papio anubis* (Olive baboon)	55.21	48.90	51.10	55.70	42.40	53.60	22.00	25.30	26.90	25.80	30.23	35.17	27.35	33.28
*Chlorocebus sabaeus* (Green monkey)	55.13	48.50	51.50	56.10	42.80	54.10	21.90	25.50	26.60	26.00	29.77	35.52	27.09	33.24
*Piliocolobus tephrosceles* (Ugandan red Colobus)	55.49	49.40	50.60	55.20	42.00	53.00	22.20	25.00	27.20	25.60	30.59	34.85	27.86	33.01
*Colobus angolensis palliatus* (Angola colobus)	54.72	48.60	51.40	55.60	42.20	55.00	22.10	25.50	26.40	26.00	29.56	36.33	26.31	33.84
*Gorilla gorilla gorilla* (Gorilla)	54.42	47.80	52.20	56.40	42.70	56.00	21.60	25.90	26.20	26.30	28.77	36.83	25.75	34.42
*Pan troglodytes* (Chimpanzee)	55.13	48.80	51.20	56.00	42.50	53.70	21.90	25.40	26.80	25.90	30.09	35.21	27.36	33.21
*Pan paniscus* (Pygmy chimpanzee)	54.85	48.60	51.40	55.80	42.40	54.40	22.00	25.50	26.60	25.90	29.81	35.77	26.73	33.64
*Homo sapiens* (Human)	55.03	48.80	51.20	55.70	42.40	53.90	22.00	25.40	26.80	25.80	29.96	35.43	27.28	33.29
*Loxodonta africana* (African savanna elephant)	55.29	49.10	50.90	55.20	42.00	53.80	22.40	25.30	26.70	25.60	30.20	35.50	27.10	33.30
*Orycteropus afer afer* (Aardvark)	54.96	48.60	51.40	55.70	42.20	54.90	22.30	25.60	26.20	25.90	29.70	36.30	26.20	33.60

*ENC represents the effective number of codons. AU represents overall A + U content. GC represents overall G + C content. GC1, GC2, and GC3 represents the frequency of the nucleotides G + C at the first, second and third positions of synonymous codons, respectively. U, C, A, and G represents the content of U, C, A, and G, respectively. U3s, C3s, A3s, and G3s represents the frequency of the nucleotides U, C, A, and G at the third position of synonymous codons, respectively. The common names of the potential hosts are provided in parenthesis.*

Effective number of codons was estimated to quantify codon usage bias among the viral coding sequences. ENC values of the complete EBOV coding sequences varied from 55.15 to 55.69 with a mean value of 55.57 ± 0.01, indicating a low codon usage bias among EBOV.

To further investigate the impact of compositional constraint on codon usage patterns of EBOV, correlation analyses of the overall base composition (A, U, G, and C%) and base composition at the third positions of synonymous codons (U3s, C3s, A3s, and G3s) with ENC were performed. ENC was noted to display significant correlation with A (*r* = −0.478, *p* < *0.01*), U (*r* = −0.409, *p* < *0.01*), C (*r* = 0.324, *p* < *0.01*), G (*r* = 0.569, *p* < *0.01*), A3s (*r* = −0.530, *p* < *0.01*), U3s (*r* = −0.547, *p* < *0.01*), C3s (*r* = 0.535, *p* < *0.01*), G3s (*r* = 0.427, *p* < *0.01*), GC (*r* = 0.629, *p* < *0.01*), and GC3 (*r* = 0.681, *p* < *0.01*) contents (Supplementary Table 2). Strong correlation of Axis 1 of the RSCU data (the principal axis of separation of genes) with GC content (*r* = 0.352, *p* < *0.01*) reinforced the impact of compositional bias on EBOV coding sequences (Supplementary Table 2).

### Relative Synonymous Codon Usage Analysis of the EBOV

A thorough Relative Synonymous Codon Usage (RSCU) analysis of the 59 codons (excluding Met, Trp, and termination codons) in EBOV coding sequences was performed to investigate the synonymous codon usage patterns of the viruses. Codons UCA (1.81) and AGA (1.78) were observed to be over-represented (RSCU ≥ 1.6) and codons UCG (0.39), ACG (0.49), GCG (0.30), CGG (0.42), and GGC (0.58) were noted as under-represented (RSCU ≤ 0.6) (Table 2). All of the over-represented codons were A-ending and all of the under-represented codons were G/C-ending (Table 2). Furthermore, a majority (18 out of 27 codons) of the preferentially used codons (RSCU > 1) were noted to be AU rich in EBOV, with 25 of the 27 preferentially employed codons ended with A/U nucleotides (Table 2). The RSCU analysis of EBOV indicated a tendency of the viral genomes toward a preference for AU rich codons over their GC rich counterparts.

**TABLE 2:**
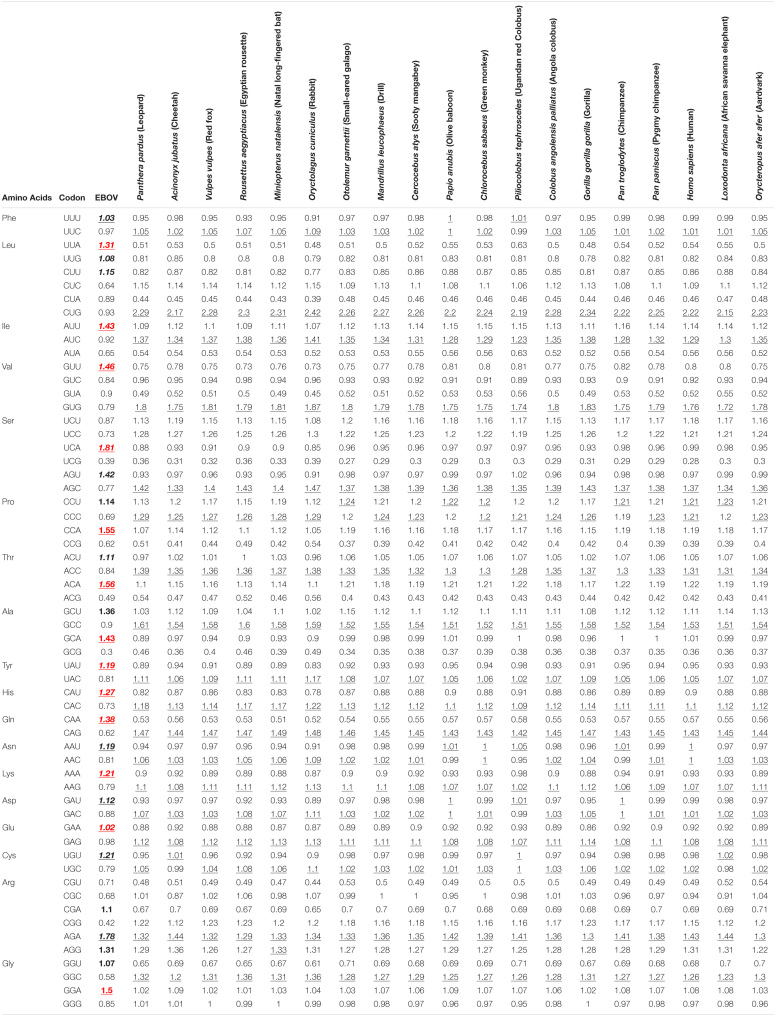
Relative synonymous codon usage (RSCU) patterns for EBOV and its potential hosts.

*Most preferred codons in the EBOV and the potential hosts are underlined; preferentially employed codons (RSCU > 1.00) in EBOV are marked in bold; AU rich preferentially employed codons in EBOV are marked in italics; most preferred codons in EBOV displaying antagonism with all potential hosts are marked in red. The common names of the potential hosts are provided in parenthesis.*

Codon usage patterns of the EBOV were meticulously compared with the codon usage profiles of its potential hosts (Table 1). It was evident that the EBOV codon usage patterns were significantly different from its potential hosts (Table 1 and Supplementary Table 3). Potential host genomes were noted to be GC rich (with average GC composition ranging between 50.60 and 53.00%), in contrast to the EBOV which exhibited an overall AU richness among its genomes (average AU composition of 56.40%) (Table 1). The ratio of coincident/antagonistic codons between EBOV and its potential hosts was observed to be *Panthera pardus* (1/17), *Acinonyx jubatus* (2/16), *Vulpes vulpes* (1/17), *Rousettus aegyptiacus* (1/17), *Miniopterus natalensis* (1/17), *Oryctolagus cuniculus* (1/17), *Otolemur garnettii* (1/17), *Mandrillus leucophaeus* (1/17), *Cercocebus atys* (1/17), *Papio anubis* (4/14), *Chlorocebus sabaeus* (2/16), *Piliocolobus tephrosceles* (5/13), *Colobus angolensis palliates* (1/17), *Gorilla gorilla gorilla* (1/17), *Pan troglodytes* (3/15), *Pan paniscus* (1/17), *Homo sapiens* (2/16), *Loxodonta Africana* (2/16), and *Orycteropus afer afer* (1/17) (Table 2), which signified an antagonistic pattern of codon usage between the EBOV and its potential hosts residing in Africa. Similar trend of antagonistic codon usage was noted when the EBOV codon usage patterns were compared with the potential mammalian hosts belonging to the order Chiroptera, Rodentia and Primates that reside in continents other than Africa (Supplementary Table 4).

### Codon Usage Bias Among EBOV Estimated From GC3-ENC and Neutrality Plots

**FIGURE 1:**
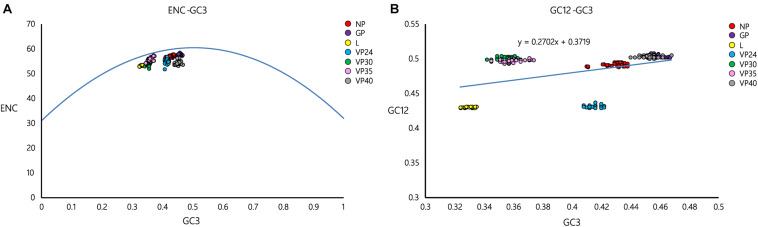
GC3-ENC and neutrality plots of EBOV. **(A)** ENC denotes the effective number of codons and GC3 denotes the GC content at the third position of synonymous codons. The solid blue line represents the ENC plot curve. EBOV genes coding for different proteins have been indicated as different colored circles. **(B)** GC12 stands for the average GC content at the first and second positions of synonymous codons, whereas, GC3 refers to the GC content at the third position of synonymous codons. EBOV genes coding for different proteins have been indicated as different colored circles. The slope of the regression line signifies the degrees of compositional bias operational on the viral genomes.

ENC values were plotted against their corresponding GC3 values for the EBOV coding sequences to estimate the effects of mutation pressure and natural selection operating on the viral genomes. A comprehensive analysis of the GC3-ENC plot (Figure 1A) for EBOV coding sequences revealed that the genes encoding for NP, GP, and VP35 clustered close to the continuous ENC plot curve whereas, the genes coding for L, VP24, VP30, and VP40 fell well below the curve.

A neutrality plot analysis was performed to identify the role of probable factors in shaping the codon usage patterns of EBOV. A thorough analysis of the neutrality plot revealed that the slope of the regression line (Figure 1B) was around 0.2702, signifying a 27.02% influence of compositional constraint on the viral coding sequences. The average translational selection index (P2) of the EBOV coding sequences was observed to be 0.51 ± 0.01.

### Differential Patterns of EBOV Adaptation in Potential Hosts

**FIGURE 2:**
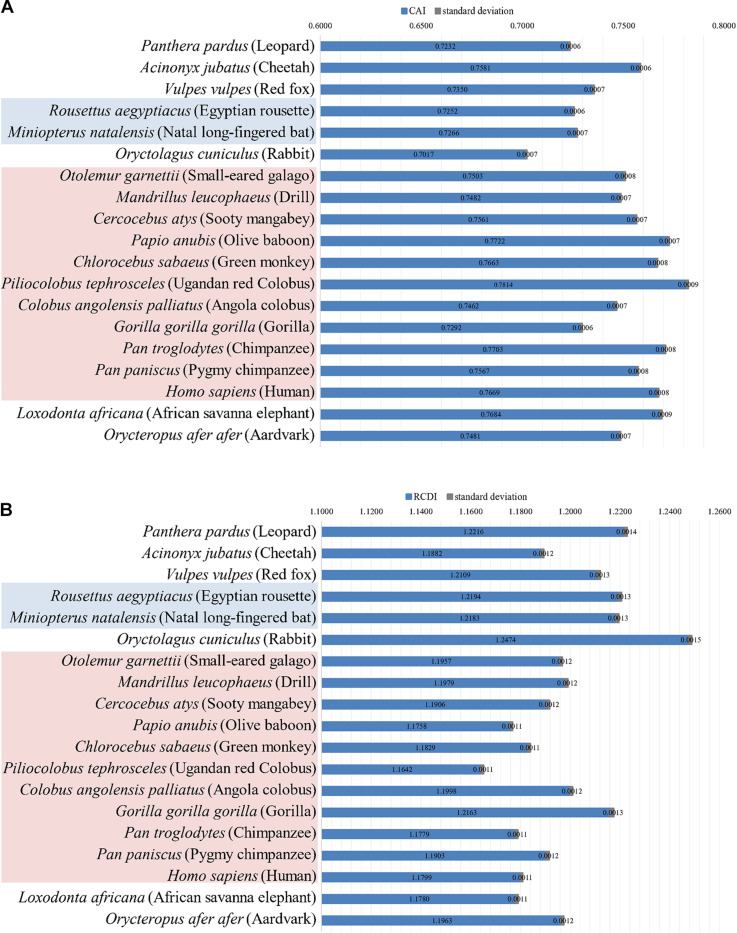
Codon adaptation index (CAI) and Relative codon deoptimization index (RCDI) of EBOV. **(A)** CAI values of the EBOV with respect to its potential hosts that dwell in Africa. Species marked with blue background are bats and species marked with red background are primates. Average CAI values and pertaining standard deviations are depicted on the histogram. **(B)** RCDI values of the EBOV with respect to their potential hosts that dwell in Africa. Species marked with blue background are bats and species marked with red background are primates. RCDI values and pertaining standard deviations are depicted on the histogram. Clustering of hosts was determined by Time Tree (http://www.timetree.org/). Bar charts were generated using the R software package.

Codon adaptation index was estimated to determine the adaptation of EBOV in its potential hosts. The average CAI values (with the standard deviations) of EBOV with respect to the different potential hosts have been depicted in Figure 2A. The highest CAI value, of 0.7814 ± 0.0009, was found between EBOV and the primate *P. tephrosceles* and the lowest CAI value, of 0.7017 ± 0.0007, was found with *O. cuniculus*. It was interesting to note that the CAI values of EBOV were significantly higher (*p* < *0.01*) for primates [except *G. g. gorilla* (0.7292 ± 0.0006)] compared to other mammals (Figure 2A). EBOV displayed significantly higher (*p* < *0.01*) CAI value with the hosts belonging to the order Primates (0.7533 ± 0.0246) in comparison to the potential hosts representing the order Rodentia (0.7230 ± 0.0295) and Chiroptera (0.7213 ± 0.0279) (Supplementary Figure 1).

Relative codon deoptimization index was estimated to further address the adaptation of EBOV in the different potential host species. EBOV displayed significantly lower (*p* < *0.01*) average RCDI value of 1.1939 ± 0.0260 with the hosts belonging to the order Primates in comparison to the potential hosts representing the order Rodentia (1.2240 ± 0.0372) and Chiroptera (1.2245 ± 0.0310) (Supplementary Figure 2). Thus, the results of CAI and RCDI analysis correlated well where EBOV displayed highest adaptation with the hosts belonging to the order Primates, as deduced from the highest CAI and lowest RCDI values (Supplementary Figures 1, 2) among the analyzed host species. On the contrary, EBOV exhibited lowest adaptation with the potential hosts representing the order Chiroptera, as evident from the lowest CAI and highest RCDI values (Supplementary Figures 1, 2) among the analyzed host species.

### Correspondence Analysis on the Basis of EBOV Codon Usage Data

**FIGURE 3:**
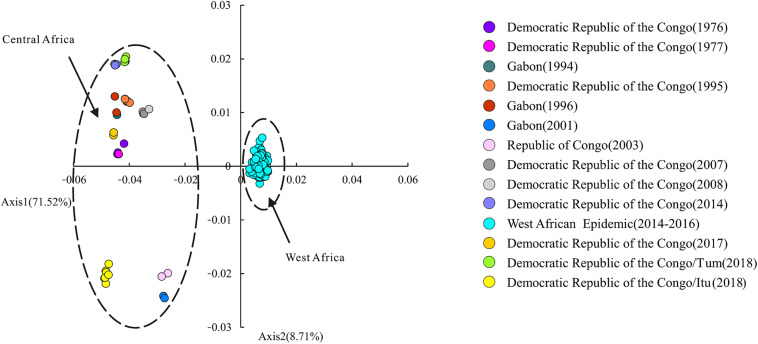
Correspondence analysis (CA) of EBOV. Correspondence analysis based on the RSCU data of EBOV variants. The position of each EBOV variant is described along the two major axes of separation, Axes 1 and 2 of the RSCU data. CA of the EBOV variants isolated from different outbreaks are depicted in different colors. “Itu” refers to the Ituri Province of Democratic Republic of the Congo; “Tum” indicates Équateur Province of Democratic Republic of the Congo.

Correspondence analysis (CA) based on the RSCU data of EBOV was performed to address the variations and trends of codon usage among the viral variants. On average, the first and second principal axes account for 71.52 and 8.71% of the total variation, respectively. The position of each EBOV variant was described along the two principal axes of separation (Figure 3).

Correspondence analysis based on the RSCU data was employed to investigate whether the EBOV variants representing different outbreaks displayed differential codon usage patterns and the CA has been depicted in Figure 3. Here, variants isolated during each outbreak have been represented in different colors (Figure 3). Variants representing the outbreaks in Gabon in 2001, Republic of Congo in 2003 and Ituri province of Democratic Republic of the Congo in 2018 fell on the lower-left half of the chart. Variants representing the other outbreaks in Central Africa clustered at the upper-left half of the chart. Variants representing the West African Epidemic (Guinea, Liberia, Mali, Nigeria and Sierra Leone) clustered along Axis 1 on the right side of the chart. It was apparent that the EBOV variants isolated from the West African Epidemic and Central African outbreaks formed two discrete clusters along Axis 1 of RSCU data. All variants representing the West African Epidemic clustered together on the right side of the Axis 1 while the variants representing the Central African outbreaks fell on the left half of Axis 1 (Figure 3). The fact that the different epidemic variants clustered separately at different positions in the chart on performing CA on RSCU data pointed toward considerable genetic diversities among them.

### Analysis of the Genetic Diversities Among EBOV

Genetic diversity among EBOV variants was analyzed with respect to the associated outbreaks. A low nucleotide diversity and high genetic identity within the viral isolates associated with a particular outbreak, revealed that each outbreak has been a single introduction of EBOV into humans (Table 3).

**TABLE 3:** Genetic diversity among EBOV variants associated with different outbreaks.

	Sequence	Number of haplotypes	Haplotype diversity	Nucleotide diversity	Genetic identity within outbreak
Total	308	246	0.995	0.00743	99.24%
Democratic Republic of the Congo (1976)	5	5	1.000	0.00051	99.95%
Democratic Republic of the Congo (1977)	1	/	/	/	/
Gabon (1994)	1	/	/	/	/
Democratic Republic of the Congo (1995)	6	4	0.867	0.00015	99.98%
Gabon (1996)	2	2	1.000	0.00304	99.69%
Gabon (2001)	2	2	1.000	0.00028	99.97%
Republic of Congo (2003)	2	2	1.000	0.00076	99.92%
Democratic Republic of the Congo (2007)	3	3	1.000	0.00028	99.97%
Democratic Republic of the Congo (2008)	1	/	/	/	/
Democratic Republic of the Congo (2014)	3	3	1.000	0.00032	99.97%
West African Epidemic (2014–2016)	264	208	0.994	0.00075	99.92%
Democratic Republic of the Congo (2017)	3	2	0.667	0.00018	99.98%
Democratic Republic of the Congo/Tum (2018)	4	4	1.000	0.00028	99.97%
Democratic Republic of the Congo/Itu (2018)	11	8	0.891	0.00020	99.98%

*“Tum” refers to Équateur Province of Democratic Republic of the Congo; “Itu” refers to the Ituri Province of Democratic Republic of the Congo.*

**FIGURE 4:**
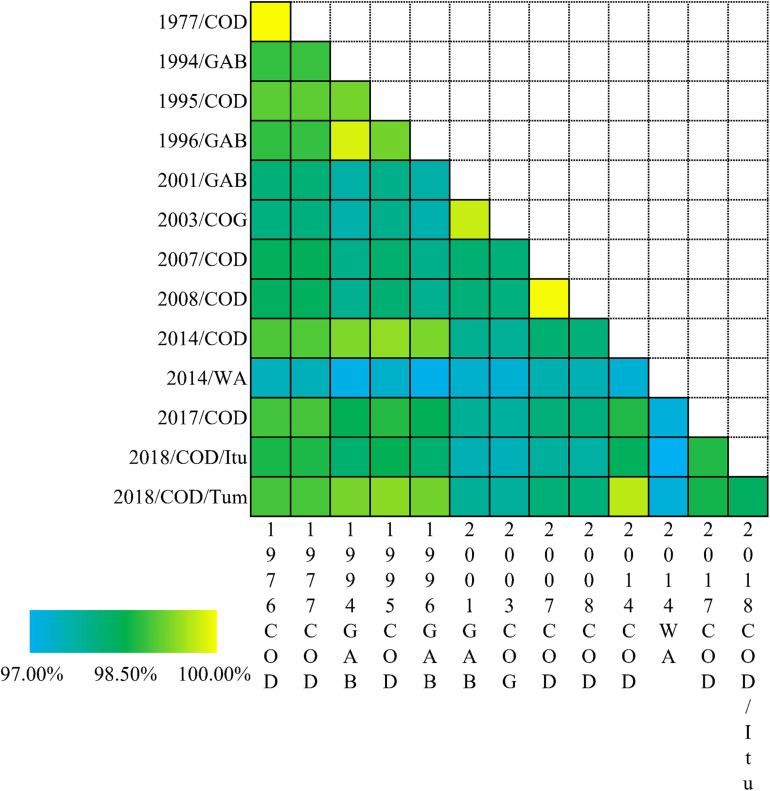
Heatmap based on the percent identities of the EBOV genomes. The percent identities of EBOV variant genomes associated with different outbreaks. COD-Democratic Republic of the Congo; GAB-Gabon; COG-Republic of the Congo. WA, West Africa Ebola epidemic; Itu-Ituri Province of Democratic Republic of the Congo; Tum-Équateur Province of Democratic Republic of the Congo.

The percentage identities of the different EBOV variants are depicted as a heatmap in Figure 4. The percentage identities of EBOV isolated from Democratic Republic of the Congo between 1976 and 1977, Gabon between 1994 and 1996 and Democratic Republic of the Congo between 2007 and 2008 were higher than the EBOV isolates associated with the West African Epidemic and other Central African outbreaks.

### Nucleotide Substitutions Among the EBOV Across Their Geographical Distributions

**FIGURE 5:**
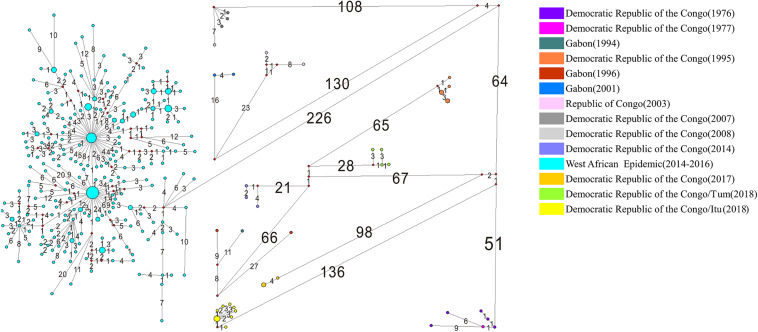
Phylogenetic networks of EBOV. Phylogenetic networks of the EBOV variants isolated from different outbreaks are depicted in different colors. The size of each node is proportional to the number of samples studied. The numbers on the lines represent the numbers of nucleotide substitutions on the lineage. “Itu” refers to the Ituri Province of Democratic Republic of the Congo; “Tum” indicates Équateur Province of Democratic Republic of the Congo.

Among the 308 EBOV genomes, 246 haplotypes were identified. The median joining network generated using the EBOV variants associated with the various outbreaks is depicted in Figure 5. The numbers on the lines in Figure 5 represent the numbers of nucleotide substitutions on the lineage and the size of the dot represents the number of variants contained in each haplotype. The haplotypes of the EBOV variants associated with the West African Epidemic were abundant because of its long-term and large-scale infection. It was evident that 226 nucleotide changes occurred between the EBOV variants associated with the West African Epidemic and other Central African outbreaks, signifying a considerable genetic divergence between them. The frequencies of nucleotide substitutions among the EBOV variants isolated from Democratic Republic of the Congo between 1976 and 1977, Gabon between 1994 and 1996 and Democratic Republic of the Congo between 2007 and 2008 were comparatively lower than the frequencies of nucleotide substitutions among the EBOV isolates representing the other Central African outbreaks.

## Discussion

Our observation (Table 1 and Table 2) of AU preference among the concerned EBOV genomes is in complete accord with a previous report (Cristina et al., 2015). AU-rich codons were preferred over their corresponding GC-rich counterparts in EBOV (Table 2). Distinct preference toward the usage of A/U was noted at the third positions of synonymous codons in EBOV (Table 2). EBOV was observed to display antagonistic codon usage patterns toward its potential hosts, as evident from a higher share of antagonistic codons over the coincident ones (Table 2). Similar instances of antagonistic codon usage by viruses relative to their hosts have been seen in the Marburg virus (Nasrullah et al., 2015) and in the hepatitis A virus (Sanchez et al., 2003). It has been inferred that this antagonism between viral and host codon usage enhances the proper folding of viral proteins, although translational efficacy might be reduced (Hu et al., 2011).

ENC values of the coding sequences of EBOV ranged from 55.15 to 55.69, indicating an overall low codon usage bias among them. Similar evidences of low codon usage bias have been observed in many RNA viruses such as hepatitis C virus (ENC, 52.62) (Hu et al., 2011), Zika virus (ZIKV) (53.93) (Butt et al., 2016) and Chikungunya virus (ENC, 55.56) (Butt et al., 2014). Our observation of low codon usage bias in EBOV appears justified in light of the fact that weak bias in codon usage in viral genomes allows reduced competition between the virus and its host for the synthesis machinery and facilitates efficient viral replication in host cells (Butt et al., 2016). It has been suggested that a virus with a low codon usage bias might be more flexible, allowing adaptation and survival in a broad range of hosts with varied codon usage patterns (Butt et al., 2016).

A detailed analysis of the GC3-ENC plot of the EBOV indicated that the viral genes encoding for NP, GP, and VP35 fell close to the continuous ENC plot curve. However, the genes encoding for the L, VP24, VP30, and VP40 clustered well below the curve (Figure 1A). It has been inferred that if the codon usage of a gene is governed only by compositional bias, then it should lie on or above the continuous ENC plot curve, whereas, the observation of genes falling well below the curve implies the roles of other factors such as natural selection, in addition to compositional constraint (Wright, 1990). Thus, apart from a subtle impact of compositional bias others factors such as natural selection have significantly influenced the codon usage in EBOV. It was noted from the neutrality plot of EBOV coding sequences that the slope of the regression line was 0.2702 (Figure 1B), which indicated that natural selection had a stronger role than compositional constraint (which contributed to only 27.02%) in shaping EBOV codon usage patterns (Nasrullah et al., 2015; Butt et al., 2016). The translational selection index (P2) > 0.50 indicates a major operational role of translational selection on the concerned genes of interest (Gatherer and McEwan, 1997). An average P2 value of 0.51 ± 0.01 for the EBOV coding sequences signified a governing role of translational selection on EBOV codon usage patterns, thus, supporting our observation from the GC3-ENC and neutrality plots. Thus, codon usage of EBOV appears to be a combined interplay of compositional bias and natural selection, with the latter being more pronounced and superior in its influence. However, previously Cristina et al. (2015) reported that mutational bias plays a major role in shaping the codon usage patterns of EBOV. The analysis by Christina and colleagues was performed on a small dataset of 25 EBOV genomes and was solely based on GC3-ENC plot to infer about the impact mutational bias on the EBOV codon usage patterns. In the present study, we have considered a larger dataset of 308 EBOV genomes and comprehensively analyzed the viral codon usage patterns employing various crucial estimates like neutrality plot and translational index (P2) apart from the GC3-ENC analysis. Similar inferences of translational selection dictating viral codon usage patterns have been previously being reported for ZIKV and Nipah virus (Butt et al., 2016; Khandia et al., 2019). The viral codon usage patterns influenced by translational selection indicate a sustained circulation of viruses in host populations and confer the viruses the ability to adapt and survive in multiple hosts (Butt et al., 2016; Khandia et al., 2019).

The host’s cellular structure and metabolism are essential for viruses to efficiently replicate and establish an infection. Codon usage patterns of viruses reflect the adaptive changes which have allowed them to optimize their survival and fitness in the host cells (Su et al., 2017; Taylor et al., 2017; Zang et al., 2017; Rahman et al., 2018; Luo et al., 2019). The CAI and RCDI are indices that can be used efficiently to analyze the adaptation of a virus to the host microcellular environment (Carbone et al., 2003; Puigbò et al., 2010). The CAI and RCDI are indices that can be used efficiently has been suggested to indicate high degrees of adaptation of a virus in a concerned host (Butt et al., 2016; Khandia et al., 2019). The present study assessed EBOV adaptation (as estimated by CAI and RCDI) to various potential hosts, where we considered a variety of African mammals whose geographical distributions overlap with EBOV, with an intent to explore viral adaptation to various mammals and further profile potential reservoirs of EBOV. It was interesting to note that EBOV displayed highest CAI value (0.7814 ± 0.0009) and lowest RCDI value (1.1642 ± 0.0011) with respect to the primate *P. tephrosceles* (Figure 2), which was consistent with the fact that EBOV has mostly been isolated from primates Gorilla (*G. g. gorilla*), Chimpanzee (*P. troglodytes*) and Human (*H*. *sapiens*) (Leroy et al., 2004). EBOV infection in the primates is frequently accompanied by severe clinical reactions possibly due to high viral adaptation with the primate expression system which might facilitate better use of host replication machinery and faster viral replication in host environment (Martines et al., 2015). However, severe clinical reactions might prove disadvantageous for long-term residence and co-existence of EBOV in primate hosts. Thus, primates show little potential to act as natural reservoirs for EBOV. Lower degrees of adaptation of emerging viruses with the natural reservoirs in comparison to the terminal hosts might facilitate long-term circulation and co-existence of the viruses in the cellular niche of natural reservoirs (Nasrullah et al., 2015). Similar instance has been reported in Marburg virus, belonging to family *Filoviridae*, where the viral pathogen has been demonstrated to adapt more efficiently with its terminal host *H. sapiens* and better utilize the translational resources compared to its natural host *R. aegyptiacus* (Nasrullah et al., 2015). African bats are speculated as the best probable candidates serving as the natural reservoirs for the ebolaviruses (Leroy et al., 2005; Pourrut et al., 2007; De Nys et al., 2018). The evidence of asymptomatic infection by EBOV was found in three species of fruit bat (*Hypsignathus monstrosus*, *Epomops franqueti*, and *Myonycteris torquata*) (Leroy et al., 2005). In the present study, it was not possible to assess EBOV adaptation and fitness in these bats due to the lack of genome sequences. However, it was interesting to note that EBOV displayed significantly lower CAI (*p* < *0.01*) values (0.7252 ± 0.0006 and 0.7266 ± 0.0007) and significantly higher (*p* < *0.01*) RCDI values (1.2194 ± 0.0013 and 1.2183 ± 0.0013) with respect to fruit bats (*R. aegyptiacus*) and insectivorous bats (*M. natalensis*), respectively, compared to the primates considered in the present analysis (Figures 2A,B). Our observations indicate toward the potential of bats to act as the natural reservoirs of EBOV. Africa exhibits a large level of biodiversity with many species yet to be discovered. It is possible that EBOV may be harbored in some unknown bat species that is yet to be characterized. In addition, it appears necessary to explore the potential of various rodents, which offer moderate adaptation to EBOV, to be involved in the transmission route of the virus (Morvan et al., 1999). Multiple crucial factors contribute to viral infection and pathogenesis in a potential host, such as species-specific interactions between the virus and host cell factors, evasion of the host immune responses, environmental and physiological factors (McElroy et al., 2018; Long et al., 2019). With the availability of more bat and mammalian genome sequences native to Africa and extensive epidemiological studies, there should be immense possibilities to accurately identify the natural reservoirs and intermediate hosts of EBOV and efficiently detect the routes of viral transmission to human population.

A thorough understanding of EBOV transmission in its terminal hosts is demanded at deeper levels. Correspondence analysis revealed that the codon usage patterns of EBOV isolates associated with the various outbreaks varied significantly. In the present study EBOV variants were classified according to the associated outbreaks based on country and year of isolation. Quite interestingly, the EBOV variants isolated from the West African outbreak and Central African outbreaks formed separate clusters on the opposite sides of Axis 1, the major axis of separation of the RSCU data (Figure 3). The separation of the Western and Central African outbreak associated EBOV isolates based on codon usage correlated well with previous phylogenetic analysis that revealed that different genetic lineages of EBOV were circulating in Central and West Africa (Holmes et al., 2016). EBOV variants isolated from Central Africa associated with different outbreaks were more dispersed than the West African Epidemic isolates (Figure 3). The EBOV isolates representing the West African Epidemic showed high haplotype diversity, moderate nucleotide diversities and higher genetic identities among them, but comparatively lower genetic identities relative to the other Central African outbreaks (Table 3 and Figure 4). Our observation was noted to be consistent with previous reports that after an initial spillover infection, the 2013–2016 EBOV outbreak in West Africa spread via chains of sustained human-to-human transmission without any additional spillover from the zoonotic reservoirs (Gire et al., 2014; Park et al., 2015). The outbreaks in Democratic Republic of the Congo between 1976 and 1977, between 1995, 2014, and 2018 (Équateur Province), between 2007 and 2008, between 2017 and 2018 (Ituri Province) were noted to show closer genetic relationship (Figure 5) and our observations appeared to be in agreement with the fact that there was more than one population of EBOV circulating in the natural reservoir of this virus and four clades of EBOV were established in the Democratic Republic of the Congo (McMullan et al., 2019).

The present study involving a robust analysis of codon usage patterns and adaptation of EBOV to diverse potential hosts dwelling in Africa promises to significantly contribute toward a better understanding of the adaptive intricacies and circulation of EBOV in various host habitats. Thus, information regarding EBOV codon usage signatures and host adaptation promise provide novel insights that could be exploited effectively to limit future cross-species transmission and spillover events from potential reservoirs to humans and arrest future outbreaks.

## Data Availability Statement

The datasets presented in this study were collected from GenBank. Accession numbers were listed in the Supplementary Table 1.

## Author Contributions

YS conceived, designed, and supervised the study. WL, AR, FG, XS, and JP generated the data. WL analyzed the data. YS, WL, and DI wrote and prepared the manuscript. All authors have read and agreed to submission of the manuscript.

## Conflict of Interest

The authors declare that the research was conducted in the absence of any commercial or financial relationships that could be construed as a potential conflict of interest.

## Footnotes

1 http://www.ncbi.nlm.nih.gov

2 https://www.ncbi.nlm.nih.gov/refseq/

3 http://sourceforge.net/projects/codonw

4 http://www.R-project.org/

5 http://www.R-project.org/

6 http://genomes.urv.cat/CAIcal/

7 http://genomes.urv.cat/CAIcal/

8 http://www.fluxus-engineering.com/sharenet.htm

## Supplementary Material

Supplementary Figure 1 Additional Codon adaptation index (CAI) analysis of EBOV. CAI values of the EBOV with respect to their potential hosts and other mammals belonging to the orders Chiroptera, Rodentia, and Primates that dwell in continents other than Africa. The mammals belonging to the orders Chiroptera, Rodentia, and Primates have been indicated with different background colors. Hosts marked in red indicate African mammals. CAI values and pertaining standard deviations are depicted on the histogram.

Supplementary Figure 2 Additional Relative codon deoptimization index (RCDI) analysis of EBOV. RCDI values of the EBOV with respect to their potential hosts and other mammals belonging to the orders Chiroptera, Rodentia, and Primates but that dwell in continents other than Africa. The mammals belong to the orders Chiroptera, Rodentia, and Primates have been indicated with different background colors. Hosts marked in red indicate African mammals. RCDI values and pertaining standard deviations are depicted on the histogram.

Supplementary Table 1 Information regarding the accession numbers, variant names, subtypes, compositional features, RSCU values, CAI values, and RCDI values of EBOV considered in the present analysis. The accession numbers for the genomic sequences of the potential host species that were used in the analysis have also been provided. The potential hosts that dwell in Africa are marked in red.

Supplementary Table 2 Correlation analysis (Spearman’s rank correlation) of the base compositional features and codon usage indices of EBOV.

Supplementary Table 3 Compositional features of EBOV and its potential hosts including mammals that belong to the orders Chiroptera, Rodentia and Primates that dwell in continents other than Africa.

Supplementary Table 4 Relative synonymous codon usage (RSCU) patterns for EBOV and its potential hosts including mammals that belong to the orders Chiroptera, Rodentia, and Primates that dwell in continents other than Africa.
