# Supplementary figures and images for: Host Adaptation and Evolutionary Analysis of Zaire ebolavirus: Insights From Codon Usage Based Investigations
Source: Front Microbiol. 2020 Nov 5;11:570131. doi: 10.3389/fmicb.2020.570131 (PMC7674656; doi:10.3389/fmicb.2020.570131)

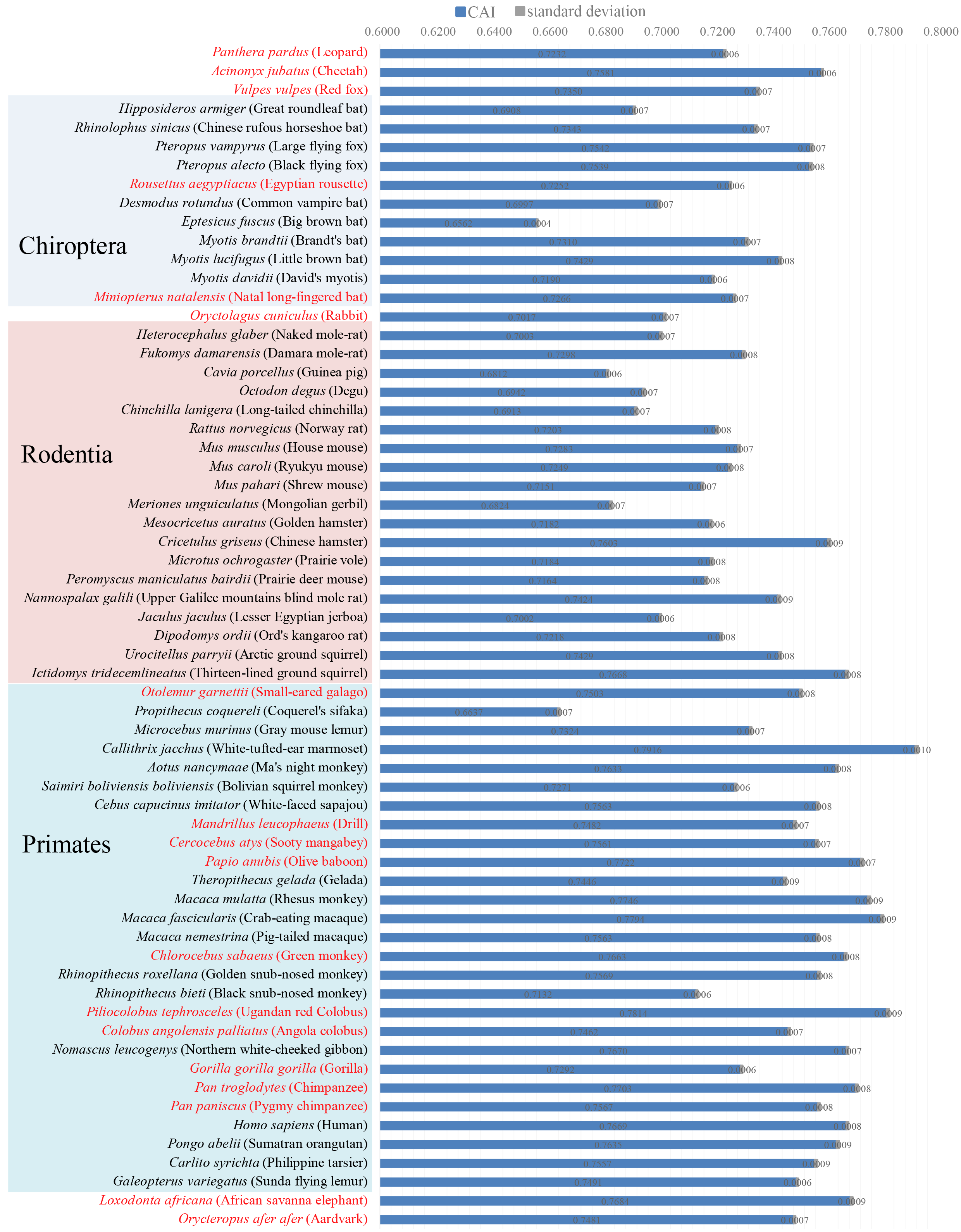

Supplement: Supplementary Figure 1 — Additional Codon adaptation index (CAI) analysis of EBOV. CAI values of the EBOV with respect to their potential hosts and other mammals belonging to the orders Chiroptera, Rodentia, and Primates that dwell in continents other than Africa. The mammals belonging to the orders Chiroptera, Rodentia, and Primates have been indicated with different background colors. Hosts marked in red indicate African mammals. CAI values and pertaining standard deviations are depicted on the histogram. [file Image_1.TIF]

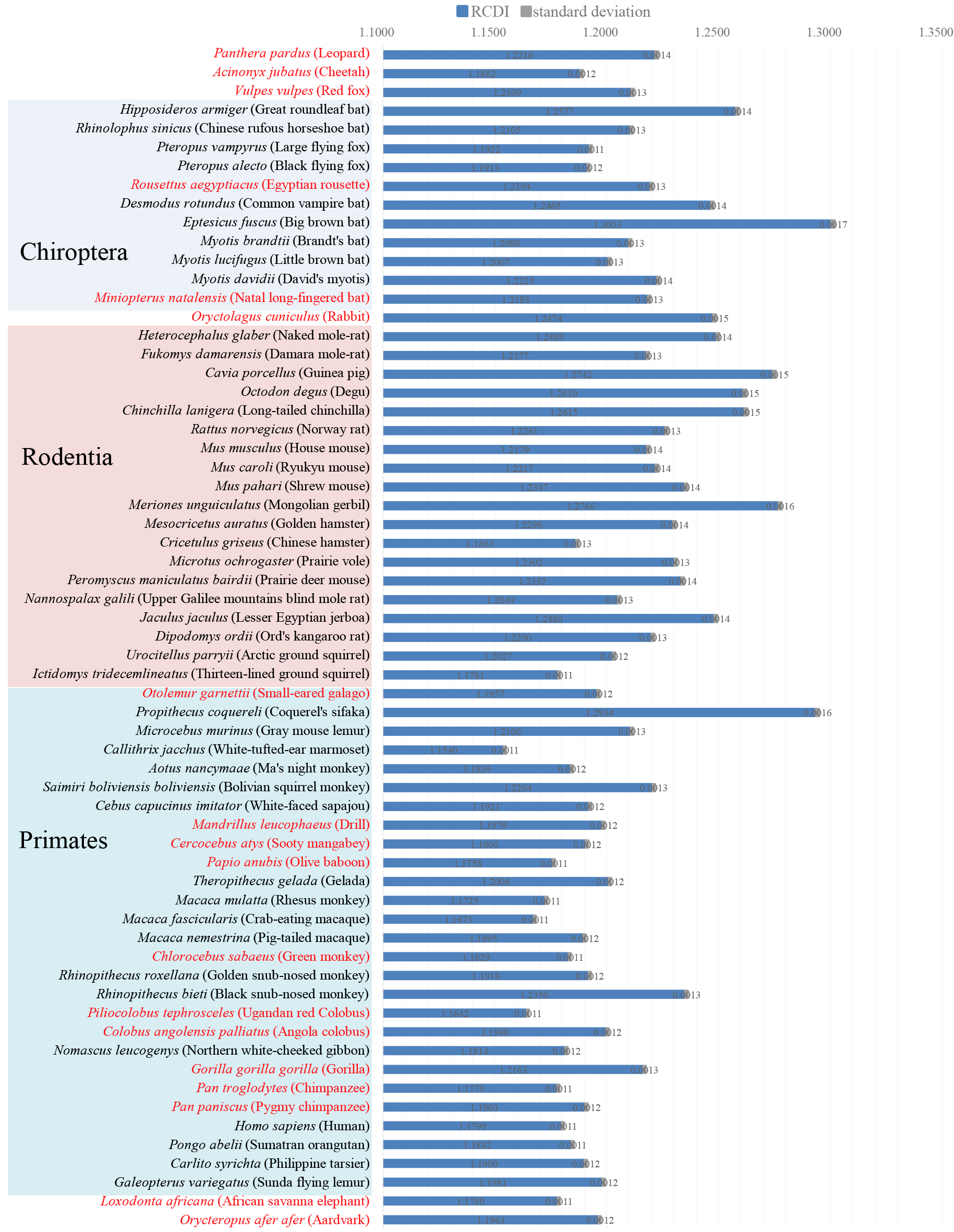

Supplement: Supplementary Figure 2 — Additional Relative codon deoptimization index (RCDI) analysis of EBOV. RCDI values of the EBOV with respect to their potential hosts and other mammals belonging to the orders Chiroptera, Rodentia, and Primates but that dwell in continents other than Africa. The mammals belong to the orders Chiroptera, Rodentia, and Primates have been indicated with different background colors. Hosts marked in red indicate African mammals. RCDI values and pertaining standard deviations are depicted on the histogram. [file Image_2.TIF]
