# Supplementary material for: Host Adaptation and Evolutionary Analysis of Zaire ebolavirus: Insights From Codon Usage Based Investigations
Source: Front Microbiol. 2020 Nov 5;11:570131. doi: 10.3389/fmicb.2020.570131 (PMC7674656; doi:10.3389/fmicb.2020.570131)
Supplement: Supplementary Table 2 — Correlation analysis (Spearman’s rank correlation) of the base compositional features and codon usage indices of EBOV. [file Table_2.docx]

**Supplementary Table 2. Correlation analysis (Spearman’s rank correlation) of the base compositional features and codon usage indices of EBOV**

|  | U | C | A | G | U3s | C3s | A3s | G3s | AU | GC | GC12 | GC3 | ENC | Axis1 (RSCU) |
| --- | --- | --- | --- | --- | --- | --- | --- | --- | --- | --- | --- | --- | --- | --- |
| C | -.295^**^ |  |  |  |  |  |  |  |  |  |  |  |  |  |
| A | .064 | -.360^**^ |  |  |  |  |  |  |  |  |  |  |  |  |
| G | -.060 | .402^**^ | -.875^**^ |  |  |  |  |  |  |  |  |  |  |  |
| U3s | .616^**^ | -.368^**^ | .344^**^ | -.401^**^ |  |  |  |  |  |  |  |  |  |  |
| C3s | -.611^**^ | .531^**^ | -.277^**^ | .305^**^ | -.770^**^ |  |  |  |  |  |  |  |  |  |
| A3s | -.006 | -.318^**^ | .578^**^ | -.570^**^ | .109 | -.263^**^ |  |  |  |  |  |  |  |  |
| G3s | .051 | .160^**^ | -.587^**^ | .598^**^ | -.127^*^ | .063 | -.718^**^ |  |  |  |  |  |  |  |
| AU | .336^**^ | -.449^**^ | .562^**^ | -.625^**^ | .483^**^ | -.473^**^ | .604^**^ | -.469^**^ |  |  |  |  |  |  |
| GC | -.336^**^ | .449^**^ | -.562^**^ | .625^**^ | -.483^**^ | .473^**^ | -.604^**^ | .469^**^ | -1.000^**^ |  |  |  |  |  |
| GC12 | .018 | .183^**^ | -.062 | .004 | .133^*^ | -.092 | -.075 | -.027 | -.166^**^ | .166^**^ |  |  |  |  |
| GC3 | -.443^**^ | .482^**^ | -.535^**^ | .580^**^ | -.697^**^ | .730^**^ | -.574^**^ | .430^**^ | -.636^**^ | .636^**^ | -.056 |  |  |  |
| ENC | -.409^**^ | .324^**^ | -.478^**^ | .569^**^ | -.547^**^ | .535^**^ | -.530^**^ | .427^**^ | -.629^**^ | .629^**^ | -.052 | .681^**^ |  |  |
| Axis1 | -.076 | .260^**^ | -.475^**^ | .512^**^ | -.373^**^ | .335^**^ | -.323^**^ | .326^**^ | -.352^**^ | .352^**^ | -.168^**^ | .436^**^ | .452^**^ |  |
| Axis2 | -.075 | .193^**^ | .248^**^ | -.223^**^ | .039 | -.039 | .103 | -.132^*^ | .104 | -.104 | .080 | -.044 | -.089 | -.215^**^ |

^**^ Correlation is significant at the 1% level of significance; ^*^ Correlation is significant at the 5% level of significance.
