# Supplementary material for: Host Adaptation and Evolutionary Analysis of Zaire ebolavirus: Insights From Codon Usage Based Investigations
Source: Front Microbiol. 2020 Nov 5;11:570131. doi: 10.3389/fmicb.2020.570131 (PMC7674656; doi:10.3389/fmicb.2020.570131)
Supplement: Supplementary Table 3 — Compositional features of EBOV and its potential hosts including mammals that belong to the orders Chiroptera, Rodentia and Primates that dwell in continents other than Africa. [file Table_3.DOCX]

| Genome | ENC | AU(%) | GC(%) | GC1(%) | GC2(%) | GC3s(%) | U(%) | C(%) | A(%) | G(%) | U3s(%) | C3s(%) | A3s(%) | G3s(%) |
| --- | --- | --- | --- | --- | --- | --- | --- | --- | --- | --- | --- | --- | --- | --- |
| EBOV | 55.57 | 56.40 | 43.60 | 51.90 | 40.40 | 38.00 | 25.40 | 23.20 | 31.10 | 20.30 | 37.30 | 25.40 | 39.80 | 23.50 |
| *Panthera pardus*(Leopard) | 54.80 | 47.50 | 52.50 | 56.40 | 42.80 | 56.70 | 21.50 | 26.00 | 26.10 | 26.40 | 28.10 | 37.30 | 25.50 | 34.90 |
| *Acinonyx jubatus*(Cheetah) | 55.14 | 48.90 | 51.10 | 55.30 | 41.90 | 54.50 | 22.40 | 25.20 | 26.60 | 25.80 | 29.80 | 35.90 | 26.70 | 33.70 |
| *Vulpes vulpes*(Red fox) | 54.75 | 47.90 | 52.10 | 56.30 | 42.70 | 55.80 | 21.70 | 25.90 | 26.20 | 26.20 | 28.90 | 36.70 | 25.80 | 34.30 |
| *Hipposideros armiger*(Great roundleaf bat) | 53.86 | 46.70 | 53.30 | 56.90 | 43.30 | 58.40 | 21.10 | 26.60 | 25.60 | 26.70 | 27.12 | 38.45 | 24.33 | 35.52 |
| *Rhinolophus sinicus*(Chinese rufous horseshoe bat) | 54.96 | 47.90 | 52.10 | 56.20 | 42.50 | 56.10 | 21.60 | 25.80 | 26.30 | 26.30 | 28.65 | 36.86 | 25.76 | 34.59 |
| *Pteropus vampyrus*(Large flying fox) | 55.20 | 48.30 | 51.70 | 56.10 | 42.80 | 54.80 | 21.90 | 25.70 | 26.40 | 26.00 | 29.45 | 36.02 | 26.51 | 33.76 |
| *Pteropus alecto*(Black flying fox) | 55.20 | 48.50 | 51.50 | 55.90 | 42.20 | 54.90 | 22.00 | 25.60 | 26.50 | 25.90 | 29.35 | 36.14 | 26.58 | 33.86 |
| *Rousettus aegyptiacus*(Egyptian rousette) | 54.79 | 47.60 | 52.40 | 56.30 | 42.80 | 56.60 | 21.60 | 26.10 | 26.10 | 26.30 | 28.13 | 37.35 | 25.52 | 34.64 |
| *Desmodus rotundus*(Common vampire bat) | 53.99 | 47.30 | 52.70 | 56.40 | 42.50 | 57.70 | 21.40 | 26.20 | 25.90 | 26.50 | 27.54 | 38.10 | 24.81 | 35.34 |
| *Eptesicus fuscus*(Big brown bat) | 53.08 | 46.20 | 53.80 | 56.90 | 42.60 | 60.50 | 21.00 | 26.80 | 25.20 | 27.00 | 25.77 | 40.07 | 23.03 | 36.84 |
| *Myotis brandtii*(Brandt's bat) | 54.67 | 48.30 | 51.70 | 55.60 | 42.10 | 55.90 | 21.70 | 25.70 | 26.60 | 26.00 | 28.69 | 37.00 | 26.03 | 34.41 |
| *Myotis lucifugus*(Little brown bat) | 54.98 | 48.40 | 51.60 | 55.80 | 42.20 | 55.40 | 21.80 | 25.60 | 26.60 | 26.00 | 29.06 | 36.46 | 26.31 | 34.32 |
| *Myotis davidii*(David's myotis) | 54.46 | 47.90 | 52.10 | 55.90 | 42.20 | 56.70 | 21.60 | 25.90 | 26.30 | 26.20 | 28.19 | 37.44 | 25.56 | 34.83 |
| *Miniopterus natalensis*(Natal long-fingered bat) | 54.52 | 48.10 | 51.90 | 56.00 | 42.40 | 56.20 | 21.80 | 25.80 | 26.20 | 26.10 | 28.79 | 36.99 | 25.56 | 34.57 |
| *Oryctolagus cuniculus*(Rabbit) | 54.15 | 47.00 | 53.00 | 56.50 | 43.20 | 57.90 | 21.20 | 26.40 | 25.80 | 26.60 | 27.10 | 38.00 | 24.90 | 35.50 |
| *Heterocephalus glaber*(Naked mole-rat) | 53.71 | 47.00 | 53.00 | 56.90 | 43.00 | 57.60 | 21.30 | 26.40 | 25.70 | 26.60 | 27.70 | 37.70 | 24.75 | 35.32 |
| *Fukomys damarensis*(Damara mole-rat) | 54.22 | 48.20 | 51.80 | 55.90 | 42.30 | 55.80 | 21.90 | 25.70 | 26.30 | 26.10 | 28.94 | 36.69 | 25.85 | 34.36 |
| *Cavia porcellus*(Guinea pig) | 53.23 | 46.70 | 53.30 | 57.00 | 43.00 | 58.60 | 21.30 | 26.60 | 25.40 | 26.70 | 27.16 | 38.42 | 23.99 | 35.83 |
| *Octodon degus*(Degu) | 53.54 | 46.90 | 53.10 | 57.00 | 43.00 | 57.90 | 21.30 | 26.50 | 25.60 | 26.60 | 27.68 | 37.95 | 24.37 | 35.44 |
| *Chinchilla lanigera*(Long-tailed chinchilla) | 53.70 | 46.70 | 53.30 | 56.80 | 43.20 | 58.40 | 21.30 | 26.50 | 25.50 | 26.70 | 27.05 | 38.18 | 24.30 | 35.81 |
| *Rattus norvegicus*(Norway rat) | 54.34 | 47.90 | 52.10 | 55.90 | 42.70 | 56.20 | 21.80 | 26.00 | 26.10 | 26.10 | 28.63 | 37.04 | 25.56 | 34.44 |
| *Mus musculus*(House mouse) | 54.42 | 48.10 | 51.90 | 55.90 | 42.70 | 55.70 | 21.80 | 25.90 | 26.30 | 26.00 | 28.96 | 36.68 | 25.88 | 34.17 |
| *Mus caroli*(Ryukyu mouse) | 54.40 | 48.10 | 51.90 | 55.80 | 42.50 | 55.90 | 22.00 | 25.90 | 26.10 | 26.00 | 28.95 | 36.88 | 25.60 | 34.22 |
| *Mus pahari*(Shrew mouse) | 54.25 | 47.80 | 52.20 | 56.00 | 42.40 | 56.60 | 21.90 | 26.00 | 25.90 | 26.20 | 28.57 | 37.40 | 25.08 | 34.62 |
| *Meriones unguiculatus*(Mongolian gerbil) | 53.47 | 46.60 | 53.40 | 57.10 | 43.20 | 58.60 | 21.20 | 26.70 | 25.30 | 26.80 | 27.25 | 38.40 | 23.90 | 35.74 |
| *Mesocricetus auratus*(Golden hamster) | 54.25 | 47.40 | 52.60 | 56.40 | 43.40 | 56.40 | 21.60 | 26.30 | 25.80 | 26.30 | 28.48 | 37.03 | 25.34 | 34.30 |
| *Cricetulus griseus*(Chinese hamster) | 54.62 | 49.40 | 50.60 | 55.20 | 41.80 | 53.10 | 22.60 | 25.10 | 26.80 | 25.50 | 30.90 | 35.02 | 27.30 | 32.84 |
| *Microtus ochrogaster*(Prairie vole) | 54.32 | 47.70 | 52.30 | 56.30 | 42.70 | 56.30 | 21.60 | 26.10 | 26.10 | 26.20 | 28.80 | 37.09 | 25.30 | 34.40 |
| *Peromyscus maniculatus bairdii*(Prairie deer mouse) | 54.22 | 47.80 | 52.20 | 56.20 | 42.60 | 56.50 | 21.50 | 26.00 | 26.30 | 26.20 | 28.55 | 37.38 | 25.39 | 34.49 |
| *Nannospalax galili*(Upper Galilee mountains blind mole rat) | 54.49 | 48.40 | 51.60 | 56.10 | 42.60 | 54.60 | 22.10 | 25.70 | 26.30 | 25.90 | 29.88 | 35.91 | 26.36 | 33.45 |
| *Jaculus jaculus*(Lesser Egyptian jerboa) | 53.99 | 47.30 | 52.70 | 56.50 | 42.70 | 57.60 | 21.60 | 26.30 | 25.60 | 26.50 | 27.96 | 38.09 | 24.44 | 35.04 |
| *Dipodomys ordii*(Ord's kangaroo rat) | 54.33 | 48.00 | 52.00 | 56.10 | 42.40 | 56.20 | 22.00 | 26.00 | 26.00 | 26.00 | 28.67 | 37.23 | 25.54 | 34.21 |
| *Urocitellus parryii*(Arctic ground squirrel) | 54.59 | 48.30 | 51.70 | 56.20 | 42.70 | 54.80 | 22.00 | 25.70 | 26.30 | 26.00 | 29.63 | 36.27 | 26.26 | 33.47 |
| *Ictidomys tridecemlineatus*(Thirteen-lined ground squirrel) | 54.96 | 49.10 | 50.90 | 55.80 | 42.10 | 53.30 | 22.30 | 25.20 | 26.90 | 25.60 | 30.71 | 35.06 | 27.27 | 32.94 |
| *Otolemur garnettii*(Small-eared galago) | 54.77 | 48.50 | 51.50 | 56.10 | 42.30 | 54.60 | 22.10 | 25.50 | 26.40 | 26.00 | 29.82 | 35.87 | 26.47 | 33.66 |
| *Propithecus coquereli*(Coquerel's sifaka) | 53.22 | 46.10 | 53.90 | 57.10 | 43.10 | 60.10 | 21.00 | 26.90 | 25.10 | 27.00 | 26.03 | 39.72 | 23.27 | 36.35 |
| *Microcebus murinus*(Gray mouse lemur) | 54.81 | 47.80 | 52.20 | 56.30 | 42.90 | 56.00 | 21.40 | 26.00 | 26.40 | 26.20 | 28.35 | 37.07 | 26.07 | 34.20 |
| *Callithrix jacchus*(White-tufted-ear marmoset) | 55.47 | 49.60 | 50.40 | 55.20 | 42.40 | 51.90 | 22.40 | 24.90 | 27.20 | 25.50 | 31.28 | 34.04 | 28.43 | 32.30 |
| *Aotus nancymaae*(Ma's night monkey) | 55.02 | 48.80 | 51.20 | 55.70 | 42.40 | 54.00 | 22.00 | 25.40 | 26.80 | 25.80 | 30.08 | 35.59 | 27.00 | 33.27 |
| *Saimiri boliviensis boliviensis*(Bolivian squirrel monkey) | 54.40 | 47.70 | 52.30 | 56.40 | 42.90 | 56.10 | 21.70 | 26.00 | 26.00 | 26.30 | 28.71 | 36.89 | 25.60 | 34.36 |
| *Cebus capucinus imitator*(White-faced sapajou) | 54.91 | 48.30 | 51.70 | 56.30 | 42.80 | 54.50 | 21.70 | 25.60 | 26.60 | 26.10 | 29.60 | 35.73 | 26.73 | 33.66 |
| *Mandrillus leucophaeus*(Drill) | 54.76 | 48.60 | 51.40 | 55.50 | 42.10 | 54.90 | 22.20 | 25.40 | 26.50 | 25.90 | 29.66 | 36.27 | 26.38 | 33.77 |
| *Cercocebus atys*(Sooty mangabey) | 54.96 | 48.40 | 51.60 | 56.10 | 42.80 | 54.60 | 21.90 | 25.70 | 26.50 | 25.90 | 29.60 | 35.86 | 26.70 | 33.57 |
| *Papio anubis*(Olive baboon) | 55.21 | 48.90 | 51.10 | 55.70 | 42.40 | 53.60 | 22.00 | 25.30 | 26.90 | 25.80 | 30.23 | 35.17 | 27.35 | 33.28 |
| *Theropithecus gelada*(Gelada) | 54.81 | 48.40 | 51.60 | 55.90 | 42.30 | 55.10 | 22.00 | 25.60 | 26.30 | 26.10 | 29.41 | 36.36 | 26.25 | 33.94 |
| *Macaca mulatta*(Rhesus monkey) | 55.27 | 49.00 | 51.00 | 55.80 | 42.30 | 53.40 | 22.00 | 25.20 | 27.00 | 25.80 | 30.39 | 35.05 | 27.53 | 33.06 |
| *Macaca fascicularis*(Crab-eating macaque) | 55.31 | 49.20 | 50.80 | 55.70 | 42.30 | 53.00 | 22.10 | 25.10 | 27.10 | 25.70 | 30.65 | 34.78 | 27.78 | 32.89 |
| *Macaca nemestrina*(Pig-tailed macaque) | 54.96 | 48.40 | 51.60 | 56.00 | 42.70 | 54.50 | 21.80 | 25.60 | 26.70 | 25.90 | 29.59 | 35.84 | 26.76 | 33.62 |
| *Chlorocebus sabaeus*(Green monkey) | 55.13 | 48.50 | 51.50 | 56.10 | 42.80 | 54.10 | 21.90 | 25.50 | 26.60 | 26.00 | 29.77 | 35.52 | 27.09 | 33.24 |
| *Rhinopithecus roxellana*(golden snub-nosed monkey) | 54.87 | 48.50 | 51.50 | 56.10 | 42.50 | 54.30 | 22.00 | 25.50 | 26.50 | 26.00 | 29.79 | 35.81 | 26.82 | 33.32 |
| *Rhinopithecus bieti*(Black snub-nosed monkey) | 54.08 | 47.40 | 52.60 | 56.60 | 43.10 | 56.90 | 21.60 | 26.20 | 25.80 | 26.40 | 28.22 | 37.41 | 25.08 | 34.80 |
| *Piliocolobus tephrosceles*(Ugandan red Colobus) | 55.49 | 49.40 | 50.60 | 55.20 | 42.00 | 53.00 | 22.20 | 25.00 | 27.20 | 25.60 | 30.59 | 34.85 | 27.86 | 33.01 |
| *Colobus angolensis palliatus*(Angola colobus) | 54.72 | 48.60 | 51.40 | 55.60 | 42.20 | 55.00 | 22.10 | 25.50 | 26.40 | 26.00 | 29.56 | 36.33 | 26.31 | 33.84 |
| *Nomascus leucogenys*(Northern white-cheeked gibbon) | 55.13 | 49.00 | 51.00 | 55.40 | 42.30 | 54.00 | 22.30 | 25.20 | 26.70 | 25.80 | 30.14 | 35.62 | 27.01 | 33.32 |
| *Gorilla gorilla gorilla*(Gorilla) | 54.42 | 47.80 | 52.20 | 56.40 | 42.70 | 56.00 | 21.60 | 25.90 | 26.20 | 26.30 | 28.77 | 36.83 | 25.75 | 34.42 |
| *Pan troglodytes*(Chimpanzee) | 55.13 | 48.80 | 51.20 | 56.00 | 42.50 | 53.70 | 21.90 | 25.40 | 26.80 | 25.90 | 30.09 | 35.21 | 27.36 | 33.21 |
| *Pan paniscus*(Pygmy chimpanzee) | 54.85 | 48.60 | 51.40 | 55.80 | 42.40 | 54.40 | 22.00 | 25.50 | 26.60 | 25.90 | 29.81 | 35.77 | 26.73 | 33.64 |
| *Homo sapiens*(Human) | 55.03 | 48.80 | 51.20 | 55.70 | 42.40 | 53.90 | 22.00 | 25.40 | 26.80 | 25.80 | 29.96 | 35.43 | 27.28 | 33.29 |
| *Pongo abelii*(Sumatran orangutan) | 55.04 | 48.80 | 51.20 | 55.80 | 42.40 | 54.10 | 22.00 | 25.40 | 26.80 | 25.80 | 29.99 | 35.57 | 27.05 | 33.40 |
| *Carlito syrichta*(Philippine tarsier) | 54.99 | 48.90 | 51.10 | 55.40 | 41.90 | 54.40 | 22.30 | 25.30 | 26.60 | 25.80 | 29.96 | 35.96 | 26.67 | 33.56 |
| *Galeopterus variegatus*(Sunda flying lemur) | 54.71 | 48.50 | 51.50 | 55.80 | 42.30 | 54.80 | 22.20 | 25.60 | 26.30 | 25.90 | 29.71 | 36.09 | 26.34 | 33.72 |
| *Loxodonta africana*(African savanna elephant) | 55.29 | 49.10 | 50.90 | 55.20 | 42.00 | 53.80 | 22.40 | 25.30 | 26.70 | 25.60 | 30.20 | 35.50 | 27.10 | 33.30 |
| *Orycteropus afer afer*(Aardvark) | 54.96 | 48.60 | 51.40 | 55.70 | 42.20 | 54.90 | 22.30 | 25.60 | 26.20 | 25.90 | 29.70 | 36.30 | 26.20 | 33.60 |

**Supplementary Table 3: Compositional features of EBOV and its potential hosts including mammals that belong to the orders Chiroptera, Rodentia and Primates that dwell in continents other than Africa.**

ENC represents the effective number of codons. AU represents overall A+U content. GC represents overall G+C content. GC1, GC2, GC3 represents the frequency of the nucleotides G+C at the first, second and third positions of synonymous codons respectively. U, C, A, G represents the content of U, C, A, and G, respectively. U3s, C3s, A3s, and G3s represents the frequency of the nucleotides U, C, A, and G at the third position of synonymous codons, respectively. The common names of the potential hosts are provided in parenthesis. Hosts marked in red indicate African mammals.
