# Supplementary material for: Host Adaptation and Evolutionary Analysis of Zaire ebolavirus: Insights From Codon Usage Based Investigations
Source: Front Microbiol. 2020 Nov 5;11:570131. doi: 10.3389/fmicb.2020.570131 (PMC7674656; doi:10.3389/fmicb.2020.570131)
Supplement: Supplementary Table 4 — Relative synonymous codon usage (RSCU) patterns for EBOV and its potential hosts including mammals that belong to the orders Chiroptera, Rodentia, and Primates that dwell in continents other than Africa. [file Table_4.DOCX]

**Supplementary Table 4: Relative synonymous codon usage (RSCU) patterns for EBOV and its potential hosts including mammals that belong to the orders Chiroptera, Rodentia and Primates that dwell in continents other than Africa.**

| Amino Acids | Phe | | Leu | | | | | | Ile | | | Val | | | | Ser | | | | | | Pro | | | | Thr | | | | Ala | | | | Tyr | | His | | Gln | | Asn | | Lys | | Asp | | Glu | | Cys | | Arg | | | | | | Gly | | | |
| --- | --- | --- | --- | --- | --- | --- | --- | --- | --- | --- | --- | --- | --- | --- | --- | --- | --- | --- | --- | --- | --- | --- | --- | --- | --- | --- | --- | --- | --- | --- | --- | --- | --- | --- | --- | --- | --- | --- | --- | --- | --- | --- | --- | --- | --- | --- | --- | --- | --- | --- | --- | --- | --- | --- | --- | --- | --- | --- | --- |
| Codon | UUU | UUC | UUA | UUG | CUU | CUC | CUA | CUG | AUU | AUC | AUA | GUU | GUC | GUA | GUG | UCU | UCC | UCA | UCG | AGU | AGC | CCU | CCC | CCA | CCG | ACU | ACC | ACA | ACG | GCU | GCC | GCA | GCG | UAU | UAC | CAU | CAC | CAA | CAG | AAU | AAC | AAA | AAG | GAU | GAC | GAA | GAG | UGU | UGC | CGU | CGC | CGA | CGG | AGA | AGG | GGU | GGC | GGA | GGG |
| EBOV | ***1.03*** | 0.97 | ***1.31*** | ***1.08*** | ***1.15*** | 0.64 | 0.89 | 0.93 | ***1.43*** | 0.92 | 0.65 | ***1.46*** | 0.84 | 0.90 | 0.79 | 0.87 | 0.73 | ***1.81*** | 0.39 | ***1.42*** | 0.77 | **1.14** | 0.69 | **1.55** | 0.62 | ***1.11*** | 0.84 | ***1.56*** | 0.49 | **1.36** | 0.90 | **1.43** | 0.30 | ***1.19*** | 0.81 | ***1.27*** | 0.73 | ***1.38*** | 0.62 | ***1.19*** | 0.81 | ***1.21*** | 0.79 | ***1.12*** | 0.88 | ***1.02*** | 0.98 | ***1.21*** | 0.79 | 0.71 | 0.68 | **1.10** | 0.42 | ***1.78*** | **1.31** | **1.07** | 0.58 | **1.50** | 0.85 |
| Panthera pardus(Leopard) | 0.95 | 1.05 | 0.51 | 0.81 | 0.82 | 1.15 | 0.44 | 2.29 | 1.09 | 1.37 | 0.54 | 0.75 | 0.96 | 0.49 | 1.80 | 1.13 | 1.28 | 0.88 | 0.36 | 0.93 | 1.42 | 1.13 | 1.29 | 1.07 | 0.51 | 0.97 | 1.39 | 1.10 | 0.54 | 1.03 | 1.61 | 0.89 | 0.46 | 0.89 | 1.11 | 0.82 | 1.18 | 0.53 | 1.47 | 0.94 | 1.06 | 0.90 | 1.10 | 0.93 | 1.07 | 0.88 | 1.12 | 0.95 | 1.05 | 0.48 | 1.01 | 0.67 | 1.22 | 1.32 | 1.29 | 0.65 | 1.32 | 1.02 | 1.01 |
| Acinonyx jubatus(Cheetah) | 0.98 | 1.02 | 0.53 | 0.85 | 0.87 | 1.14 | 0.45 | 2.17 | 1.12 | 1.34 | 0.54 | 0.78 | 0.95 | 0.52 | 1.75 | 1.19 | 1.27 | 0.93 | 0.31 | 0.97 | 1.33 | 1.20 | 1.25 | 1.14 | 0.41 | 1.02 | 1.35 | 1.15 | 0.47 | 1.12 | 1.54 | 0.97 | 0.36 | 0.94 | 1.06 | 0.87 | 1.13 | 0.56 | 1.44 | 0.97 | 1.03 | 0.92 | 1.08 | 0.97 | 1.03 | 0.92 | 1.08 | 1.01 | 0.99 | 0.51 | 0.87 | 0.70 | 1.12 | 1.44 | 1.36 | 0.69 | 1.20 | 1.09 | 1.01 |
| Vulpes vulpes(Red fox) | 0.95 | 1.05 | 0.50 | 0.80 | 0.82 | 1.14 | 0.45 | 2.28 | 1.10 | 1.37 | 0.53 | 0.75 | 0.94 | 0.51 | 1.81 | 1.15 | 1.26 | 0.91 | 0.32 | 0.96 | 1.40 | 1.17 | 1.27 | 1.12 | 0.44 | 1.01 | 1.36 | 1.16 | 0.47 | 1.09 | 1.58 | 0.94 | 0.40 | 0.91 | 1.09 | 0.86 | 1.14 | 0.53 | 1.47 | 0.97 | 1.03 | 0.89 | 1.11 | 0.97 | 1.03 | 0.88 | 1.12 | 0.96 | 1.04 | 0.49 | 1.02 | 0.69 | 1.23 | 1.32 | 1.26 | 0.67 | 1.31 | 1.02 | 1.00 |
| Hipposideros armiger(Great roundleaf bat) | 0.91 | 1.09 | 0.46 | 0.77 | 0.76 | 1.16 | 0.42 | 2.44 | 1.06 | 1.44 | 0.50 | 0.69 | 0.98 | 0.46 | 1.87 | 1.06 | 1.30 | 0.87 | 0.37 | 0.91 | 1.50 | 1.12 | 1.32 | 1.07 | 0.48 | 0.95 | 1.42 | 1.11 | 0.52 | 1.03 | 1.64 | 0.89 | 0.45 | 0.85 | 1.15 | 0.80 | 1.20 | 0.50 | 1.50 | 0.90 | 1.10 | 0.86 | 1.14 | 0.88 | 1.12 | 0.83 | 1.17 | 0.92 | 1.08 | 0.48 | 1.09 | 0.67 | 1.29 | 1.20 | 1.26 | 0.63 | 1.38 | 0.96 | 1.03 |
| Rhinolophus sinicus(Chinese rufous horseshoe bat) | 0.94 | 1.06 | 0.51 | 0.82 | 0.82 | 1.14 | 0.43 | 2.28 | 1.10 | 1.36 | 0.53 | 0.77 | 0.96 | 0.48 | 1.79 | 1.12 | 1.25 | 0.90 | 0.37 | 0.96 | 1.40 | 1.16 | 1.25 | 1.12 | 0.47 | 0.99 | 1.35 | 1.13 | 0.53 | 1.10 | 1.55 | 0.93 | 0.43 | 0.87 | 1.13 | 0.84 | 1.16 | 0.54 | 1.46 | 0.94 | 1.06 | 0.90 | 1.10 | 0.93 | 1.07 | 0.88 | 1.12 | 0.96 | 1.04 | 0.51 | 1.01 | 0.68 | 1.21 | 1.31 | 1.27 | 0.68 | 1.29 | 1.03 | 0.99 |
| Pteropus vampyrus(Large flying fox) | 0.96 | 1.04 | 0.54 | 0.83 | 0.84 | 1.11 | 0.47 | 2.22 | 1.12 | 1.32 | 0.56 | 0.77 | 0.94 | 0.54 | 1.76 | 1.17 | 1.20 | 0.95 | 0.33 | 0.97 | 1.38 | 1.19 | 1.23 | 1.15 | 0.44 | 1.06 | 1.32 | 1.17 | 0.46 | 1.10 | 1.55 | 0.95 | 0.40 | 0.93 | 1.07 | 0.87 | 1.13 | 0.54 | 1.46 | 0.99 | 1.01 | 0.91 | 1.09 | 0.96 | 1.04 | 0.90 | 1.10 | 0.97 | 1.03 | 0.52 | 0.99 | 0.70 | 1.19 | 1.34 | 1.25 | 0.68 | 1.30 | 1.04 | 0.99 |
| Pteropus alecto(Black flying fox) | 0.96 | 1.04 | 0.54 | 0.83 | 0.84 | 1.11 | 0.46 | 2.22 | 1.12 | 1.32 | 0.56 | 0.77 | 0.95 | 0.53 | 1.75 | 1.16 | 1.21 | 0.94 | 0.33 | 0.97 | 1.38 | 1.18 | 1.23 | 1.14 | 0.44 | 1.04 | 1.32 | 1.17 | 0.47 | 1.10 | 1.55 | 0.95 | 0.40 | 0.93 | 1.07 | 0.87 | 1.13 | 0.55 | 1.45 | 0.98 | 1.02 | 0.92 | 1.08 | 0.96 | 1.04 | 0.91 | 1.09 | 0.96 | 1.04 | 0.51 | 0.98 | 0.70 | 1.18 | 1.37 | 1.27 | 0.67 | 1.30 | 1.05 | 0.98 |
| Rousettus aegyptiacus(Egyptian rousette) | 0.93 | 1.07 | 0.51 | 0.80 | 0.81 | 1.14 | 0.44 | 2.30 | 1.09 | 1.38 | 0.54 | 0.73 | 0.98 | 0.50 | 1.79 | 1.13 | 1.25 | 0.90 | 0.36 | 0.93 | 1.43 | 1.15 | 1.26 | 1.10 | 0.49 | 1.00 | 1.36 | 1.13 | 0.52 | 1.04 | 1.60 | 0.90 | 0.46 | 0.89 | 1.11 | 0.83 | 1.17 | 0.53 | 1.47 | 0.95 | 1.05 | 0.89 | 1.11 | 0.92 | 1.08 | 0.88 | 1.12 | 0.92 | 1.08 | 0.49 | 1.06 | 0.67 | 1.23 | 1.29 | 1.27 | 0.65 | 1.36 | 1.01 | 0.99 |
| Desmodus rotundus(Common vampire bat) | 0.91 | 1.09 | 0.47 | 0.78 | 0.78 | 1.15 | 0.41 | 2.40 | 1.06 | 1.42 | 0.51 | 0.72 | 0.97 | 0.46 | 1.86 | 1.10 | 1.30 | 0.87 | 0.35 | 0.94 | 1.44 | 1.15 | 1.29 | 1.10 | 0.45 | 0.98 | 1.41 | 1.11 | 0.51 | 1.07 | 1.60 | 0.92 | 0.41 | 0.82 | 1.18 | 0.78 | 1.22 | 0.50 | 1.50 | 0.90 | 1.10 | 0.87 | 1.13 | 0.88 | 1.12 | 0.85 | 1.15 | 0.91 | 1.09 | 0.47 | 1.01 | 0.68 | 1.24 | 1.28 | 1.32 | 0.65 | 1.33 | 0.99 | 1.03 |
| Eptesicus fuscus(Big brown bat) | 0.86 | 1.14 | 0.45 | 0.74 | 0.71 | 1.21 | 0.37 | 2.52 | 1.03 | 1.49 | 0.48 | 0.67 | 0.99 | 0.42 | 1.92 | 1.02 | 1.39 | 0.80 | 0.41 | 0.87 | 1.51 | 1.05 | 1.41 | 0.98 | 0.57 | 0.90 | 1.48 | 1.02 | 0.59 | 0.96 | 1.71 | 0.81 | 0.52 | 0.81 | 1.19 | 0.75 | 1.25 | 0.49 | 1.51 | 0.87 | 1.13 | 0.83 | 1.17 | 0.85 | 1.15 | 0.81 | 1.19 | 0.85 | 1.15 | 0.43 | 1.17 | 0.64 | 1.36 | 1.15 | 1.25 | 0.59 | 1.44 | 0.94 | 1.04 |
| Myotis brandtii(Brandt's bat) | 0.95 | 1.05 | 0.53 | 0.82 | 0.81 | 1.15 | 0.43 | 2.28 | 1.10 | 1.37 | 0.53 | 0.76 | 0.95 | 0.48 | 1.81 | 1.13 | 1.30 | 0.90 | 0.33 | 0.96 | 1.38 | 1.17 | 1.28 | 1.12 | 0.43 | 0.99 | 1.38 | 1.14 | 0.49 | 1.09 | 1.57 | 0.95 | 0.38 | 0.89 | 1.11 | 0.84 | 1.16 | 0.54 | 1.46 | 0.94 | 1.06 | 0.90 | 1.10 | 0.94 | 1.06 | 0.89 | 1.11 | 0.95 | 1.05 | 0.47 | 0.94 | 0.70 | 1.20 | 1.37 | 1.33 | 0.66 | 1.28 | 1.06 | 1.00 |
| Myotis lucifugus(Little brown bat) | 0.96 | 1.04 | 0.54 | 0.83 | 0.82 | 1.12 | 0.43 | 2.25 | 1.12 | 1.33 | 0.55 | 0.78 | 0.94 | 0.50 | 1.79 | 1.14 | 1.26 | 0.91 | 0.34 | 0.97 | 1.38 | 1.17 | 1.26 | 1.11 | 0.46 | 1.02 | 1.35 | 1.15 | 0.49 | 1.09 | 1.55 | 0.94 | 0.42 | 0.91 | 1.09 | 0.85 | 1.15 | 0.55 | 1.45 | 0.96 | 1.04 | 0.91 | 1.09 | 0.95 | 1.05 | 0.91 | 1.09 | 0.95 | 1.05 | 0.46 | 0.96 | 0.70 | 1.20 | 1.38 | 1.30 | 0.66 | 1.28 | 1.06 | 1.00 |
| Myotis davidii(David's myotis) | 0.93 | 1.07 | 0.51 | 0.81 | 0.79 | 1.15 | 0.42 | 2.32 | 1.09 | 1.38 | 0.52 | 0.74 | 0.96 | 0.48 | 1.82 | 1.12 | 1.31 | 0.88 | 0.34 | 0.94 | 1.41 | 1.15 | 1.30 | 1.10 | 0.45 | 0.97 | 1.39 | 1.13 | 0.51 | 1.07 | 1.60 | 0.93 | 0.41 | 0.88 | 1.12 | 0.82 | 1.18 | 0.53 | 1.47 | 0.93 | 1.07 | 0.89 | 1.11 | 0.92 | 1.08 | 0.88 | 1.12 | 0.93 | 1.07 | 0.46 | 0.97 | 0.68 | 1.23 | 1.33 | 1.32 | 0.65 | 1.30 | 1.04 | 1.01 |
| Miniopterus natalensis(Natal long-fingered bat) | 0.95 | 1.05 | 0.51 | 0.80 | 0.82 | 1.12 | 0.43 | 2.31 | 1.11 | 1.36 | 0.53 | 0.76 | 0.94 | 0.49 | 1.81 | 1.15 | 1.26 | 0.90 | 0.33 | 0.95 | 1.40 | 1.19 | 1.28 | 1.12 | 0.42 | 1.03 | 1.37 | 1.14 | 0.46 | 1.10 | 1.58 | 0.93 | 0.39 | 0.89 | 1.11 | 0.83 | 1.17 | 0.51 | 1.49 | 0.94 | 1.06 | 0.88 | 1.12 | 0.93 | 1.07 | 0.87 | 1.13 | 0.94 | 1.06 | 0.47 | 0.98 | 0.69 | 1.20 | 1.33 | 1.33 | 0.67 | 1.31 | 1.03 | 1.00 |
| Oryctolagus cuniculus(Rabbit) | 0.91 | 1.09 | 0.48 | 0.79 | 0.77 | 1.15 | 0.39 | 2.42 | 1.07 | 1.41 | 0.52 | 0.73 | 0.96 | 0.45 | 1.87 | 1.08 | 1.30 | 0.85 | 0.39 | 0.91 | 1.47 | 1.12 | 1.29 | 1.05 | 0.54 | 0.96 | 1.38 | 1.10 | 0.56 | 1.02 | 1.59 | 0.90 | 0.49 | 0.83 | 1.17 | 0.78 | 1.22 | 0.52 | 1.48 | 0.91 | 1.09 | 0.87 | 1.13 | 0.89 | 1.11 | 0.87 | 1.13 | 0.90 | 1.10 | 0.44 | 1.07 | 0.65 | 1.20 | 1.34 | 1.31 | 0.61 | 1.36 | 1.04 | 0.99 |
| Heterocephalus glaber(Naked mole-rat) | 0.91 | 1.09 | 0.44 | 0.76 | 0.77 | 1.14 | 0.43 | 2.46 | 1.06 | 1.44 | 0.51 | 0.70 | 0.94 | 0.47 | 1.89 | 1.10 | 1.28 | 0.89 | 0.32 | 0.94 | 1.48 | 1.16 | 1.29 | 1.11 | 0.44 | 1.01 | 1.38 | 1.18 | 0.43 | 1.07 | 1.59 | 0.94 | 0.40 | 0.86 | 1.14 | 0.81 | 1.19 | 0.50 | 1.50 | 0.94 | 1.06 | 0.84 | 1.16 | 0.92 | 1.08 | 0.83 | 1.17 | 0.91 | 1.09 | 0.44 | 1.06 | 0.69 | 1.24 | 1.25 | 1.32 | 0.65 | 1.36 | 0.98 | 1.01 |
| Fukomys damarensis(Damara mole-rat) | 0.93 | 1.07 | 0.48 | 0.79 | 0.81 | 1.12 | 0.44 | 2.35 | 1.08 | 1.39 | 0.53 | 0.73 | 0.94 | 0.50 | 1.83 | 1.16 | 1.27 | 0.91 | 0.28 | 0.96 | 1.41 | 1.22 | 1.23 | 1.16 | 0.38 | 1.04 | 1.35 | 1.20 | 0.41 | 1.14 | 1.53 | 0.99 | 0.33 | 0.88 | 1.12 | 0.83 | 1.17 | 0.52 | 1.48 | 0.96 | 1.04 | 0.87 | 1.13 | 0.94 | 1.06 | 0.87 | 1.13 | 0.95 | 1.05 | 0.48 | 0.96 | 0.69 | 1.15 | 1.35 | 1.36 | 0.69 | 1.28 | 1.05 | 0.98 |
| Cavia porcellus(Guinea pig) | 0.88 | 1.12 | 0.41 | 0.74 | 0.74 | 1.15 | 0.42 | 2.54 | 1.03 | 1.50 | 0.48 | 0.65 | 0.96 | 0.46 | 1.93 | 1.08 | 1.31 | 0.85 | 0.32 | 0.92 | 1.50 | 1.17 | 1.29 | 1.10 | 0.44 | 0.99 | 1.42 | 1.14 | 0.45 | 1.07 | 1.59 | 0.94 | 0.40 | 0.83 | 1.17 | 0.79 | 1.21 | 0.48 | 1.52 | 0.90 | 1.10 | 0.82 | 1.18 | 0.89 | 1.11 | 0.80 | 1.20 | 0.91 | 1.09 | 0.47 | 1.11 | 0.68 | 1.25 | 1.20 | 1.29 | 0.66 | 1.38 | 0.96 | 1.01 |
| Octodon degus(Degu) | 0.89 | 1.11 | 0.42 | 0.75 | 0.76 | 1.14 | 0.43 | 2.48 | 1.06 | 1.46 | 0.49 | 0.68 | 0.94 | 0.47 | 1.90 | 1.11 | 1.28 | 0.87 | 0.32 | 0.93 | 1.50 | 1.20 | 1.25 | 1.13 | 0.42 | 1.00 | 1.38 | 1.17 | 0.44 | 1.09 | 1.56 | 0.94 | 0.40 | 0.84 | 1.16 | 0.80 | 1.20 | 0.48 | 1.52 | 0.91 | 1.09 | 0.82 | 1.18 | 0.91 | 1.09 | 0.81 | 1.19 | 0.91 | 1.09 | 0.48 | 1.12 | 0.67 | 1.25 | 1.21 | 1.27 | 0.66 | 1.39 | 0.96 | 0.99 |
| Chinchilla lanigera(Long-tailed chinchilla) | 0.89 | 1.11 | 0.45 | 0.75 | 0.76 | 1.16 | 0.41 | 2.48 | 1.04 | 1.45 | 0.51 | 0.69 | 0.96 | 0.46 | 1.89 | 1.08 | 1.30 | 0.85 | 0.34 | 0.92 | 1.50 | 1.15 | 1.29 | 1.06 | 0.50 | 0.97 | 1.39 | 1.13 | 0.50 | 1.05 | 1.57 | 0.92 | 0.45 | 0.83 | 1.17 | 0.78 | 1.22 | 0.49 | 1.51 | 0.90 | 1.10 | 0.84 | 1.16 | 0.89 | 1.11 | 0.82 | 1.18 | 0.90 | 1.10 | 0.45 | 1.09 | 0.64 | 1.24 | 1.26 | 1.32 | 0.64 | 1.36 | 0.98 | 1.02 |
| Rattus norvegicus(Norway rat) | 0.90 | 1.10 | 0.44 | 0.81 | 0.81 | 1.16 | 0.49 | 2.29 | 1.03 | 1.45 | 0.51 | 0.72 | 0.98 | 0.50 | 1.80 | 1.17 | 1.26 | 0.91 | 0.29 | 0.96 | 1.41 | 1.26 | 1.17 | 1.18 | 0.39 | 1.02 | 1.34 | 1.21 | 0.43 | 1.17 | 1.50 | 0.98 | 0.36 | 0.85 | 1.15 | 0.83 | 1.17 | 0.52 | 1.48 | 0.88 | 1.12 | 0.82 | 1.18 | 0.89 | 1.11 | 0.83 | 1.17 | 0.98 | 1.02 | 0.51 | 0.92 | 0.73 | 1.11 | 1.36 | 1.37 | 0.71 | 1.27 | 1.06 | 0.96 |
| Mus musculus(House mouse) | 0.92 | 1.08 | 0.45 | 0.81 | 0.82 | 1.15 | 0.49 | 2.28 | 1.04 | 1.44 | 0.52 | 0.73 | 0.98 | 0.50 | 1.79 | 1.18 | 1.25 | 0.91 | 0.29 | 0.96 | 1.41 | 1.26 | 1.15 | 1.20 | 0.38 | 1.02 | 1.33 | 1.22 | 0.42 | 1.19 | 1.48 | 0.99 | 0.34 | 0.87 | 1.13 | 0.85 | 1.15 | 0.53 | 1.47 | 0.89 | 1.11 | 0.83 | 1.17 | 0.92 | 1.08 | 0.84 | 1.16 | 0.99 | 1.01 | 0.49 | 0.91 | 0.73 | 1.11 | 1.38 | 1.37 | 0.71 | 1.27 | 1.07 | 0.95 |
| Mus caroli(Ryukyu mouse) | 0.91 | 1.09 | 0.44 | 0.81 | 0.82 | 1.15 | 0.49 | 2.30 | 1.05 | 1.44 | 0.51 | 0.72 | 0.97 | 0.50 | 1.80 | 1.18 | 1.25 | 0.90 | 0.30 | 0.95 | 1.42 | 1.26 | 1.16 | 1.19 | 0.39 | 1.03 | 1.34 | 1.22 | 0.41 | 1.18 | 1.49 | 0.97 | 0.35 | 0.88 | 1.12 | 0.85 | 1.15 | 0.53 | 1.47 | 0.89 | 1.11 | 0.82 | 1.18 | 0.92 | 1.08 | 0.84 | 1.16 | 0.98 | 1.02 | 0.50 | 0.96 | 0.74 | 1.12 | 1.35 | 1.32 | 0.71 | 1.28 | 1.06 | 0.95 |
| Mus pahari(Shrew mouse) | 0.90 | 1.10 | 0.43 | 0.80 | 0.80 | 1.17 | 0.48 | 2.33 | 1.04 | 1.47 | 0.49 | 0.71 | 0.98 | 0.49 | 1.82 | 1.16 | 1.27 | 0.87 | 0.31 | 0.95 | 1.43 | 1.23 | 1.19 | 1.16 | 0.42 | 1.01 | 1.36 | 1.20 | 0.43 | 1.16 | 1.51 | 0.95 | 0.38 | 0.87 | 1.13 | 0.84 | 1.16 | 0.51 | 1.49 | 0.88 | 1.12 | 0.81 | 1.19 | 0.91 | 1.09 | 0.83 | 1.17 | 0.97 | 1.03 | 0.51 | 0.99 | 0.74 | 1.15 | 1.31 | 1.31 | 0.70 | 1.30 | 1.04 | 0.95 |
| Meriones unguiculatus(Mongolian gerbil) | 0.88 | 1.12 | 0.39 | 0.76 | 0.78 | 1.18 | 0.44 | 2.46 | 1.02 | 1.50 | 0.48 | 0.68 | 0.97 | 0.46 | 1.88 | 1.12 | 1.30 | 0.86 | 0.32 | 0.90 | 1.50 | 1.20 | 1.24 | 1.10 | 0.46 | 0.97 | 1.39 | 1.16 | 0.48 | 1.10 | 1.58 | 0.90 | 0.42 | 0.81 | 1.19 | 0.79 | 1.21 | 0.47 | 1.53 | 0.85 | 1.15 | 0.79 | 1.21 | 0.88 | 1.12 | 0.80 | 1.20 | 0.92 | 1.08 | 0.47 | 1.06 | 0.70 | 1.22 | 1.22 | 1.33 | 0.66 | 1.38 | 0.99 | 0.98 |
| Mesocricetus auratus(Golden hamster) | 0.90 | 1.10 | 0.42 | 0.81 | 0.81 | 1.15 | 0.47 | 2.33 | 1.04 | 1.45 | 0.51 | 0.72 | 0.97 | 0.50 | 1.82 | 1.15 | 1.26 | 0.91 | 0.30 | 0.95 | 1.41 | 1.23 | 1.20 | 1.17 | 0.40 | 1.02 | 1.35 | 1.22 | 0.41 | 1.15 | 1.52 | 0.97 | 0.36 | 0.86 | 1.14 | 0.85 | 1.15 | 0.52 | 1.48 | 0.89 | 1.11 | 0.82 | 1.18 | 0.90 | 1.10 | 0.83 | 1.17 | 0.96 | 1.04 | 0.50 | 0.97 | 0.72 | 1.13 | 1.34 | 1.34 | 0.70 | 1.29 | 1.04 | 0.96 |
| Cricetulus griseus(Chinese hamster) | 0.96 | 1.04 | 0.47 | 0.86 | 0.88 | 1.09 | 0.52 | 2.18 | 1.11 | 1.36 | 0.53 | 0.78 | 0.93 | 0.54 | 1.75 | 1.24 | 1.21 | 0.98 | 0.23 | 1.02 | 1.33 | 1.32 | 1.13 | 1.27 | 0.28 | 1.11 | 1.28 | 1.31 | 0.31 | 1.26 | 1.44 | 1.06 | 0.24 | 0.93 | 1.07 | 0.92 | 1.08 | 0.56 | 1.44 | 0.96 | 1.04 | 0.86 | 1.14 | 0.98 | 1.02 | 0.90 | 1.10 | 1.04 | 0.96 | 0.53 | 0.83 | 0.77 | 1.04 | 1.46 | 1.37 | 0.76 | 1.20 | 1.11 | 0.93 |
| Microtus ochrogaster(Prairie vole) | 0.90 | 1.10 | 0.43 | 0.81 | 0.81 | 1.15 | 0.47 | 2.33 | 1.05 | 1.45 | 0.49 | 0.73 | 0.97 | 0.49 | 1.81 | 1.17 | 1.27 | 0.88 | 0.31 | 0.95 | 1.42 | 1.26 | 1.18 | 1.16 | 0.40 | 1.01 | 1.36 | 1.19 | 0.44 | 1.17 | 1.50 | 0.96 | 0.36 | 0.86 | 1.14 | 0.84 | 1.16 | 0.51 | 1.49 | 0.89 | 1.11 | 0.82 | 1.18 | 0.92 | 1.08 | 0.85 | 1.15 | 0.97 | 1.03 | 0.52 | 0.99 | 0.73 | 1.16 | 1.30 | 1.29 | 0.71 | 1.29 | 1.05 | 0.95 |
| Peromyscus maniculatus bairdii(Prairie deer mouse) | 0.90 | 1.10 | 0.44 | 0.79 | 0.81 | 1.17 | 0.45 | 2.35 | 1.06 | 1.44 | 0.50 | 0.73 | 1.00 | 0.48 | 1.80 | 1.14 | 1.29 | 0.87 | 0.32 | 0.96 | 1.42 | 1.23 | 1.20 | 1.16 | 0.41 | 1.01 | 1.37 | 1.18 | 0.44 | 1.14 | 1.55 | 0.94 | 0.37 | 0.85 | 1.15 | 0.85 | 1.15 | 0.51 | 1.49 | 0.90 | 1.10 | 0.84 | 1.16 | 0.91 | 1.09 | 0.86 | 1.14 | 0.98 | 1.02 | 0.49 | 0.96 | 0.71 | 1.16 | 1.37 | 1.32 | 0.70 | 1.29 | 1.05 | 0.96 |
| Nannospalax galili(Upper Galilee mountains blind mole rat) | 0.94 | 1.06 | 0.46 | 0.82 | 0.84 | 1.10 | 0.50 | 2.28 | 1.10 | 1.40 | 0.50 | 0.75 | 0.92 | 0.53 | 1.80 | 1.19 | 1.24 | 0.95 | 0.25 | 0.99 | 1.37 | 1.27 | 1.17 | 1.22 | 0.33 | 1.07 | 1.34 | 1.25 | 0.34 | 1.18 | 1.49 | 1.01 | 0.31 | 0.92 | 1.08 | 0.89 | 1.11 | 0.53 | 1.47 | 0.95 | 1.05 | 0.86 | 1.14 | 0.97 | 1.03 | 0.87 | 1.13 | 0.99 | 1.01 | 0.53 | 0.96 | 0.75 | 1.13 | 1.33 | 1.29 | 0.73 | 1.26 | 1.06 | 0.94 |
| Jaculus jaculus(Lesser Egyptian jerboa) | 0.90 | 1.10 | 0.43 | 0.80 | 0.79 | 1.15 | 0.44 | 2.38 | 1.06 | 1.46 | 0.48 | 0.70 | 0.96 | 0.46 | 1.88 | 1.11 | 1.30 | 0.86 | 0.33 | 0.93 | 1.46 | 1.19 | 1.26 | 1.12 | 0.43 | 0.99 | 1.40 | 1.14 | 0.47 | 1.10 | 1.59 | 0.90 | 0.41 | 0.86 | 1.14 | 0.82 | 1.18 | 0.51 | 1.49 | 0.90 | 1.10 | 0.82 | 1.18 | 0.89 | 1.11 | 0.83 | 1.17 | 0.93 | 1.07 | 0.51 | 1.13 | 0.68 | 1.21 | 1.22 | 1.26 | 0.69 | 1.35 | 1.01 | 0.95 |
| Dipodomys ordii(Ord's kangaroo rat) | 0.92 | 1.08 | 0.48 | 0.80 | 0.80 | 1.14 | 0.46 | 2.32 | 1.09 | 1.42 | 0.50 | 0.71 | 0.92 | 0.51 | 1.85 | 1.17 | 1.29 | 0.89 | 0.30 | 0.94 | 1.42 | 1.19 | 1.27 | 1.13 | 0.41 | 1.00 | 1.39 | 1.17 | 0.43 | 1.11 | 1.59 | 0.91 | 0.38 | 0.89 | 1.11 | 0.85 | 1.15 | 0.55 | 1.45 | 0.96 | 1.04 | 0.87 | 1.13 | 0.96 | 1.04 | 0.88 | 1.12 | 0.95 | 1.05 | 0.48 | 1.06 | 0.70 | 1.21 | 1.31 | 1.24 | 0.67 | 1.32 | 1.04 | 0.96 |
| Urocitellus parryii(Arctic ground squirrel) | 0.95 | 1.05 | 0.49 | 0.82 | 0.84 | 1.11 | 0.46 | 2.29 | 1.12 | 1.36 | 0.52 | 0.76 | 0.92 | 0.51 | 1.81 | 1.17 | 1.26 | 0.93 | 0.28 | 0.98 | 1.38 | 1.23 | 1.22 | 1.18 | 0.36 | 1.06 | 1.37 | 1.19 | 0.38 | 1.13 | 1.55 | 0.97 | 0.34 | 0.93 | 1.07 | 0.87 | 1.13 | 0.55 | 1.45 | 0.98 | 1.02 | 0.89 | 1.11 | 0.98 | 1.02 | 0.89 | 1.11 | 0.96 | 1.04 | 0.51 | 1.03 | 0.71 | 1.17 | 1.33 | 1.26 | 0.70 | 1.29 | 1.05 | 0.96 |
| Ictidomys tridecemlineatus(Thirteen-lined ground squirrel) | 0.99 | 1.01 | 0.53 | 0.86 | 0.87 | 1.07 | 0.49 | 2.19 | 1.15 | 1.31 | 0.54 | 0.79 | 0.90 | 0.54 | 1.77 | 1.22 | 1.22 | 0.97 | 0.25 | 1.01 | 1.32 | 1.27 | 1.19 | 1.22 | 0.32 | 1.09 | 1.33 | 1.22 | 0.36 | 1.18 | 1.50 | 1.02 | 0.30 | 0.96 | 1.04 | 0.91 | 1.09 | 0.57 | 1.43 | 1.01 | 0.99 | 0.91 | 1.09 | 1.01 | 0.99 | 0.91 | 1.09 | 1.00 | 1.00 | 0.51 | 0.97 | 0.73 | 1.12 | 1.39 | 1.29 | 0.73 | 1.23 | 1.10 | 0.94 |
| Otolemur garnettii(Small-eared galago) | 0.97 | 1.03 | 0.51 | 0.82 | 0.83 | 1.09 | 0.48 | 2.26 | 1.12 | 1.35 | 0.53 | 0.75 | 0.93 | 0.52 | 1.80 | 1.20 | 1.22 | 0.96 | 0.27 | 0.98 | 1.37 | 1.24 | 1.20 | 1.19 | 0.37 | 1.06 | 1.33 | 1.21 | 0.40 | 1.15 | 1.52 | 0.99 | 0.34 | 0.92 | 1.08 | 0.87 | 1.13 | 0.54 | 1.46 | 0.98 | 1.02 | 0.90 | 1.10 | 0.97 | 1.03 | 0.89 | 1.11 | 0.98 | 1.02 | 0.53 | 0.99 | 0.70 | 1.18 | 1.33 | 1.27 | 0.71 | 1.28 | 1.03 | 0.98 |
| Propithecus coquereli(Coquerel's sifaka) | 0.87 | 1.13 | 0.42 | 0.74 | 0.73 | 1.20 | 0.40 | 2.51 | 1.02 | 1.50 | 0.48 | 0.66 | 1.00 | 0.43 | 1.91 | 1.05 | 1.35 | 0.83 | 0.39 | 0.86 | 1.53 | 1.08 | 1.37 | 1.02 | 0.53 | 0.94 | 1.45 | 1.06 | 0.55 | 0.99 | 1.68 | 0.84 | 0.49 | 0.81 | 1.19 | 0.75 | 1.25 | 0.49 | 1.51 | 0.87 | 1.13 | 0.83 | 1.17 | 0.85 | 1.15 | 0.80 | 1.20 | 0.86 | 1.14 | 0.46 | 1.19 | 0.64 | 1.34 | 1.15 | 1.23 | 0.60 | 1.45 | 0.92 | 1.03 |
| Microcebus murinus(Gray mouse lemur) | 0.94 | 1.06 | 0.52 | 0.79 | 0.82 | 1.12 | 0.45 | 2.31 | 1.09 | 1.35 | 0.55 | 0.77 | 0.95 | 0.51 | 1.78 | 1.13 | 1.25 | 0.91 | 0.35 | 0.93 | 1.43 | 1.16 | 1.25 | 1.11 | 0.48 | 1.02 | 1.34 | 1.14 | 0.49 | 1.05 | 1.58 | 0.93 | 0.45 | 0.89 | 1.11 | 0.83 | 1.17 | 0.55 | 1.45 | 0.95 | 1.05 | 0.92 | 1.08 | 0.93 | 1.07 | 0.89 | 1.11 | 0.92 | 1.08 | 0.47 | 1.05 | 0.65 | 1.21 | 1.34 | 1.28 | 0.64 | 1.35 | 1.02 | 0.99 |
| Callithrix jacchus(White-tufted-ear marmoset) | 1.03 | 0.97 | 0.58 | 0.85 | 0.92 | 1.06 | 0.48 | 2.11 | 1.19 | 1.23 | 0.58 | 0.84 | 0.89 | 0.56 | 1.71 | 1.22 | 1.17 | 1.01 | 0.27 | 1.01 | 1.32 | 1.25 | 1.14 | 1.23 | 0.38 | 1.10 | 1.26 | 1.25 | 0.39 | 1.16 | 1.44 | 1.05 | 0.34 | 0.98 | 1.02 | 0.94 | 1.06 | 0.59 | 1.41 | 1.03 | 0.97 | 0.96 | 1.04 | 1.03 | 0.97 | 0.96 | 1.04 | 1.01 | 0.99 | 0.50 | 0.89 | 0.72 | 1.07 | 1.52 | 1.31 | 0.72 | 1.20 | 1.15 | 0.94 |
| Aotus nancymaae(Ma's night monkey) | 0.99 | 1.01 | 0.54 | 0.81 | 0.87 | 1.09 | 0.46 | 2.23 | 1.15 | 1.31 | 0.54 | 0.79 | 0.91 | 0.53 | 1.77 | 1.18 | 1.21 | 0.97 | 0.28 | 0.99 | 1.37 | 1.22 | 1.21 | 1.18 | 0.39 | 1.06 | 1.32 | 1.20 | 0.42 | 1.12 | 1.53 | 1.00 | 0.36 | 0.94 | 1.06 | 0.90 | 1.10 | 0.56 | 1.44 | 0.99 | 1.01 | 0.92 | 1.08 | 0.99 | 1.01 | 0.91 | 1.09 | 0.98 | 1.02 | 0.50 | 0.97 | 0.71 | 1.16 | 1.40 | 1.26 | 0.69 | 1.27 | 1.08 | 0.96 |
| Saimiri boliviensis boliviensis(Bolivian squirrel monkey) | 0.95 | 1.05 | 0.48 | 0.78 | 0.82 | 1.13 | 0.43 | 2.36 | 1.11 | 1.38 | 0.51 | 0.74 | 0.94 | 0.49 | 1.82 | 1.14 | 1.26 | 0.92 | 0.31 | 0.94 | 1.43 | 1.18 | 1.26 | 1.14 | 0.43 | 1.01 | 1.37 | 1.16 | 0.46 | 1.09 | 1.58 | 0.96 | 0.38 | 0.90 | 1.10 | 0.85 | 1.15 | 0.52 | 1.48 | 0.96 | 1.04 | 0.88 | 1.12 | 0.95 | 1.05 | 0.86 | 1.14 | 0.94 | 1.06 | 0.50 | 1.03 | 0.70 | 1.23 | 1.29 | 1.26 | 0.67 | 1.31 | 1.03 | 0.99 |
| Cebus capucinus imitator(White-faced sapajou) | 0.98 | 1.02 | 0.52 | 0.80 | 0.85 | 1.09 | 0.45 | 2.28 | 1.15 | 1.31 | 0.54 | 0.79 | 0.91 | 0.52 | 1.78 | 1.17 | 1.22 | 0.96 | 0.30 | 0.97 | 1.40 | 1.20 | 1.21 | 1.17 | 0.42 | 1.05 | 1.31 | 1.20 | 0.43 | 1.10 | 1.53 | 0.98 | 0.39 | 0.94 | 1.06 | 0.88 | 1.12 | 0.55 | 1.45 | 0.99 | 1.01 | 0.92 | 1.08 | 0.98 | 1.02 | 0.90 | 1.10 | 0.96 | 1.04 | 0.50 | 1.00 | 0.69 | 1.19 | 1.36 | 1.26 | 0.68 | 1.30 | 1.05 | 0.97 |
| Mandrillus leucophaeus(Drill) | 0.97 | 1.03 | 0.50 | 0.81 | 0.85 | 1.13 | 0.45 | 2.27 | 1.13 | 1.34 | 0.53 | 0.77 | 0.93 | 0.51 | 1.79 | 1.16 | 1.25 | 0.95 | 0.29 | 0.97 | 1.38 | 1.21 | 1.24 | 1.16 | 0.39 | 1.05 | 1.35 | 1.18 | 0.43 | 1.12 | 1.55 | 0.98 | 0.35 | 0.93 | 1.07 | 0.88 | 1.12 | 0.55 | 1.45 | 0.98 | 1.02 | 0.90 | 1.10 | 0.98 | 1.02 | 0.89 | 1.11 | 0.97 | 1.03 | 0.50 | 1.00 | 0.70 | 1.16 | 1.36 | 1.28 | 0.69 | 1.27 | 1.07 | 0.98 |
| Cercocebus atys(Sooty mangabey) | 0.98 | 1.02 | 0.52 | 0.81 | 0.86 | 1.10 | 0.46 | 2.26 | 1.14 | 1.31 | 0.55 | 0.78 | 0.92 | 0.52 | 1.78 | 1.16 | 1.23 | 0.96 | 0.30 | 0.97 | 1.39 | 1.20 | 1.23 | 1.16 | 0.42 | 1.05 | 1.32 | 1.19 | 0.43 | 1.10 | 1.54 | 0.99 | 0.38 | 0.93 | 1.07 | 0.88 | 1.12 | 0.55 | 1.45 | 0.99 | 1.01 | 0.92 | 1.08 | 0.98 | 1.02 | 0.90 | 1.10 | 0.98 | 1.02 | 0.49 | 1.00 | 0.69 | 1.18 | 1.35 | 1.27 | 0.68 | 1.29 | 1.06 | 0.97 |
| Papio anubis(Olive baboon) | 1.00 | 1.00 | 0.55 | 0.83 | 0.88 | 1.08 | 0.46 | 2.20 | 1.15 | 1.28 | 0.56 | 0.81 | 0.91 | 0.53 | 1.75 | 1.18 | 1.20 | 0.97 | 0.29 | 0.99 | 1.36 | 1.22 | 1.20 | 1.18 | 0.41 | 1.07 | 1.30 | 1.21 | 0.42 | 1.12 | 1.51 | 1.01 | 0.37 | 0.95 | 1.05 | 0.90 | 1.10 | 0.57 | 1.43 | 1.01 | 0.99 | 0.93 | 1.07 | 1.00 | 1.00 | 0.92 | 1.08 | 0.99 | 1.01 | 0.49 | 0.95 | 0.70 | 1.15 | 1.42 | 1.29 | 0.69 | 1.25 | 1.09 | 0.96 |
| Theropithecus gelada(Gelada) | 0.96 | 1.04 | 0.51 | 0.81 | 0.85 | 1.11 | 0.45 | 2.29 | 1.13 | 1.35 | 0.52 | 0.77 | 0.92 | 0.51 | 1.80 | 1.15 | 1.24 | 0.94 | 0.31 | 0.97 | 1.40 | 1.18 | 1.24 | 1.15 | 0.43 | 1.04 | 1.35 | 1.17 | 0.44 | 1.09 | 1.55 | 0.96 | 0.39 | 0.92 | 1.08 | 0.88 | 1.12 | 0.55 | 1.45 | 0.98 | 1.02 | 0.90 | 1.10 | 0.97 | 1.03 | 0.89 | 1.11 | 0.96 | 1.04 | 0.50 | 1.04 | 0.69 | 1.19 | 1.34 | 1.24 | 0.68 | 1.30 | 1.05 | 0.96 |
| *Macaca mulatta*(Rhesus monkey) | 1.00 | 1.00 | 0.55 | 0.83 | 0.88 | 1.07 | 0.47 | 2.20 | 1.16 | 1.28 | 0.56 | 0.81 | 0.90 | 0.54 | 1.74 | 1.19 | 1.20 | 0.98 | 0.29 | 1.00 | 1.35 | 1.22 | 1.18 | 1.19 | 0.40 | 1.08 | 1.29 | 1.22 | 0.42 | 1.12 | 1.50 | 1.01 | 0.36 | 0.95 | 1.05 | 0.91 | 1.09 | 0.57 | 1.43 | 1.01 | 0.99 | 0.94 | 1.06 | 1.01 | 0.99 | 0.93 | 1.07 | 0.99 | 1.01 | 0.50 | 0.97 | 0.71 | 1.15 | 1.42 | 1.26 | 0.70 | 1.26 | 1.09 | 0.95 |
| Macaca fascicularis(Crab-eating macaque) | 1.00 | 1.00 | 0.56 | 0.84 | 0.89 | 1.07 | 0.47 | 2.17 | 1.17 | 1.26 | 0.57 | 0.82 | 0.90 | 0.54 | 1.73 | 1.20 | 1.19 | 0.99 | 0.28 | 1.00 | 1.34 | 1.24 | 1.18 | 1.20 | 0.39 | 1.09 | 1.28 | 1.22 | 0.41 | 1.14 | 1.50 | 1.02 | 0.35 | 0.96 | 1.04 | 0.91 | 1.09 | 0.58 | 1.42 | 1.02 | 0.98 | 0.95 | 1.05 | 1.01 | 0.99 | 0.94 | 1.06 | 1.00 | 1.00 | 0.50 | 0.94 | 0.71 | 1.13 | 1.44 | 1.28 | 0.71 | 1.24 | 1.10 | 0.95 |
| Macaca nemestrina(Pig-tailed macaque) | 0.98 | 1.02 | 0.53 | 0.81 | 0.85 | 1.10 | 0.46 | 2.25 | 1.13 | 1.31 | 0.55 | 0.78 | 0.91 | 0.52 | 1.79 | 1.16 | 1.23 | 0.96 | 0.30 | 0.97 | 1.39 | 1.20 | 1.22 | 1.16 | 0.42 | 1.05 | 1.32 | 1.19 | 0.44 | 1.10 | 1.54 | 0.99 | 0.37 | 0.93 | 1.07 | 0.88 | 1.12 | 0.56 | 1.44 | 0.99 | 1.01 | 0.92 | 1.08 | 0.98 | 1.02 | 0.90 | 1.10 | 0.97 | 1.03 | 0.49 | 0.99 | 0.69 | 1.18 | 1.37 | 1.29 | 0.68 | 1.28 | 1.07 | 0.97 |
| Chlorocebus sabaeus(Green monkey) | 0.98 | 1.02 | 0.53 | 0.81 | 0.87 | 1.10 | 0.46 | 2.24 | 1.15 | 1.29 | 0.56 | 0.80 | 0.91 | 0.53 | 1.75 | 1.16 | 1.22 | 0.97 | 0.30 | 0.97 | 1.38 | 1.20 | 1.20 | 1.17 | 0.42 | 1.06 | 1.30 | 1.21 | 0.43 | 1.10 | 1.52 | 0.99 | 0.39 | 0.94 | 1.06 | 0.88 | 1.12 | 0.57 | 1.43 | 1.00 | 1.00 | 0.93 | 1.07 | 0.99 | 1.01 | 0.92 | 1.08 | 0.97 | 1.03 | 0.50 | 1.00 | 0.68 | 1.16 | 1.39 | 1.27 | 0.69 | 1.27 | 1.07 | 0.97 |
| Rhinopithecus roxellana(Golden snub-nosed monkey) | 0.96 | 1.04 | 0.51 | 0.80 | 0.85 | 1.11 | 0.46 | 2.28 | 1.15 | 1.32 | 0.53 | 0.80 | 0.91 | 0.52 | 1.76 | 1.17 | 1.23 | 0.96 | 0.29 | 0.97 | 1.38 | 1.22 | 1.20 | 1.19 | 0.39 | 1.06 | 1.32 | 1.20 | 0.41 | 1.12 | 1.52 | 0.99 | 0.36 | 0.93 | 1.07 | 0.88 | 1.12 | 0.55 | 1.45 | 0.99 | 1.01 | 0.93 | 1.07 | 0.99 | 1.01 | 0.91 | 1.09 | 0.96 | 1.04 | 0.51 | 1.01 | 0.69 | 1.17 | 1.36 | 1.24 | 0.70 | 1.28 | 1.07 | 0.95 |
| Rhinopithecus bieti(Black snub-nosed monkey) | 0.93 | 1.07 | 0.45 | 0.77 | 0.79 | 1.16 | 0.43 | 2.39 | 1.08 | 1.41 | 0.51 | 0.72 | 0.95 | 0.48 | 1.85 | 1.11 | 1.29 | 0.91 | 0.31 | 0.93 | 1.45 | 1.18 | 1.27 | 1.13 | 0.42 | 1.01 | 1.39 | 1.15 | 0.45 | 1.07 | 1.60 | 0.95 | 0.39 | 0.89 | 1.11 | 0.84 | 1.16 | 0.51 | 1.49 | 0.94 | 1.06 | 0.86 | 1.14 | 0.93 | 1.07 | 0.84 | 1.16 | 0.93 | 1.07 | 0.48 | 1.05 | 0.67 | 1.24 | 1.26 | 1.29 | 0.65 | 1.32 | 1.01 | 1.01 |
| Piliocolobus tephrosceles(Ugandan red Colobus) | 1.01 | 0.99 | 0.63 | 0.81 | 0.85 | 1.06 | 0.46 | 2.19 | 1.15 | 1.23 | 0.63 | 0.81 | 0.89 | 0.56 | 1.74 | 1.17 | 1.19 | 0.97 | 0.30 | 1.02 | 1.35 | 1.20 | 1.21 | 1.17 | 0.42 | 1.07 | 1.28 | 1.22 | 0.43 | 1.11 | 1.51 | 1.00 | 0.38 | 0.98 | 1.02 | 0.91 | 1.09 | 0.58 | 1.42 | 1.05 | 0.95 | 0.98 | 1.02 | 1.01 | 0.99 | 0.93 | 1.07 | 1.00 | 1.00 | 0.50 | 0.98 | 0.69 | 1.16 | 1.41 | 1.25 | 0.71 | 1.26 | 1.07 | 0.95 |
| Colobus angolensis palliatus(Angola colobus) | 0.97 | 1.03 | 0.50 | 0.80 | 0.85 | 1.12 | 0.45 | 2.28 | 1.13 | 1.35 | 0.52 | 0.77 | 0.93 | 0.50 | 1.80 | 1.15 | 1.25 | 0.95 | 0.29 | 0.96 | 1.39 | 1.20 | 1.24 | 1.16 | 0.40 | 1.05 | 1.35 | 1.18 | 0.43 | 1.11 | 1.55 | 0.98 | 0.36 | 0.93 | 1.07 | 0.88 | 1.12 | 0.55 | 1.45 | 0.98 | 1.02 | 0.90 | 1.10 | 0.97 | 1.03 | 0.89 | 1.11 | 0.97 | 1.03 | 0.50 | 1.01 | 0.69 | 1.17 | 1.36 | 1.28 | 0.69 | 1.28 | 1.06 | 0.98 |
| Nomascus leucogenys(Northern white-cheeked gibbon) | 0.99 | 1.01 | 0.53 | 0.82 | 0.87 | 1.11 | 0.46 | 2.21 | 1.16 | 1.30 | 0.55 | 0.80 | 0.92 | 0.52 | 1.76 | 1.17 | 1.23 | 0.97 | 0.29 | 0.97 | 1.36 | 1.20 | 1.22 | 1.17 | 0.41 | 1.07 | 1.32 | 1.20 | 0.41 | 1.12 | 1.51 | 0.99 | 0.38 | 0.95 | 1.05 | 0.91 | 1.09 | 0.57 | 1.43 | 1.00 | 1.00 | 0.93 | 1.07 | 0.99 | 1.01 | 0.92 | 1.08 | 0.98 | 1.02 | 0.50 | 0.99 | 0.68 | 1.13 | 1.41 | 1.29 | 0.69 | 1.26 | 1.08 | 0.97 |
| Gorilla gorilla gorilla(Gorilla) | 0.95 | 1.05 | 0.48 | 0.78 | 0.81 | 1.13 | 0.44 | 2.34 | 1.11 | 1.38 | 0.52 | 0.75 | 0.93 | 0.49 | 1.83 | 1.13 | 1.26 | 0.93 | 0.31 | 0.94 | 1.43 | 1.17 | 1.26 | 1.15 | 0.42 | 1.02 | 1.37 | 1.17 | 0.44 | 1.08 | 1.58 | 0.96 | 0.38 | 0.91 | 1.09 | 0.86 | 1.14 | 0.53 | 1.47 | 0.96 | 1.04 | 0.88 | 1.12 | 0.95 | 1.05 | 0.86 | 1.14 | 0.94 | 1.06 | 0.49 | 1.03 | 0.68 | 1.23 | 1.30 | 1.28 | 0.67 | 1.31 | 1.02 | 1.00 |
| Pan troglodytes(Chimpanzee) | 0.99 | 1.01 | 0.54 | 0.82 | 0.87 | 1.08 | 0.46 | 2.22 | 1.16 | 1.28 | 0.56 | 0.82 | 0.90 | 0.53 | 1.75 | 1.17 | 1.20 | 0.98 | 0.29 | 0.98 | 1.37 | 1.21 | 1.19 | 1.19 | 0.40 | 1.07 | 1.30 | 1.22 | 0.42 | 1.12 | 1.52 | 1.00 | 0.37 | 0.95 | 1.05 | 0.89 | 1.11 | 0.57 | 1.43 | 1.01 | 0.99 | 0.94 | 1.06 | 1.00 | 1.00 | 0.92 | 1.08 | 0.98 | 1.02 | 0.49 | 0.96 | 0.69 | 1.17 | 1.41 | 1.28 | 0.69 | 1.27 | 1.08 | 0.97 |
| Pan paniscus(Pygmy chimpanzee) | 0.98 | 1.02 | 0.52 | 0.81 | 0.85 | 1.10 | 0.46 | 2.25 | 1.14 | 1.32 | 0.54 | 0.78 | 0.91 | 0.52 | 1.79 | 1.17 | 1.22 | 0.96 | 0.29 | 0.98 | 1.38 | 1.21 | 1.23 | 1.18 | 0.39 | 1.06 | 1.33 | 1.19 | 0.42 | 1.12 | 1.54 | 1.00 | 0.35 | 0.94 | 1.06 | 0.89 | 1.11 | 0.55 | 1.45 | 0.99 | 1.01 | 0.91 | 1.09 | 0.99 | 1.01 | 0.90 | 1.10 | 0.98 | 1.02 | 0.49 | 0.97 | 0.70 | 1.17 | 1.38 | 1.29 | 0.68 | 1.27 | 1.07 | 0.98 |
| Homo sapiens(Human) | 0.99 | 1.01 | 0.54 | 0.82 | 0.86 | 1.09 | 0.46 | 2.22 | 1.14 | 1.29 | 0.56 | 0.80 | 0.92 | 0.52 | 1.76 | 1.18 | 1.21 | 0.99 | 0.28 | 0.97 | 1.37 | 1.21 | 1.21 | 1.19 | 0.39 | 1.05 | 1.31 | 1.22 | 0.42 | 1.11 | 1.53 | 1.01 | 0.36 | 0.95 | 1.05 | 0.90 | 1.10 | 0.57 | 1.43 | 1.00 | 1.00 | 0.93 | 1.07 | 0.99 | 1.01 | 0.92 | 1.08 | 0.98 | 1.02 | 0.49 | 0.94 | 0.69 | 1.15 | 1.43 | 1.31 | 0.68 | 1.26 | 1.08 | 0.97 |
| Pongo abelii(Sumatran orangutan) | 0.98 | 1.02 | 0.53 | 0.82 | 0.86 | 1.10 | 0.46 | 2.23 | 1.14 | 1.31 | 0.55 | 0.80 | 0.91 | 0.53 | 1.77 | 1.17 | 1.21 | 0.97 | 0.29 | 0.98 | 1.38 | 1.21 | 1.21 | 1.18 | 0.40 | 1.06 | 1.31 | 1.21 | 0.42 | 1.11 | 1.52 | 0.99 | 0.37 | 0.95 | 1.05 | 0.90 | 1.10 | 0.56 | 1.44 | 1.00 | 1.00 | 0.92 | 1.08 | 0.99 | 1.01 | 0.92 | 1.08 | 0.98 | 1.02 | 0.49 | 0.98 | 0.69 | 1.16 | 1.40 | 1.27 | 0.68 | 1.27 | 1.07 | 0.97 |
| Carlito syrichta(Philippine tarsier) | 0.98 | 1.02 | 0.51 | 0.83 | 0.87 | 1.11 | 0.45 | 2.23 | 1.14 | 1.34 | 0.52 | 0.78 | 0.94 | 0.51 | 1.77 | 1.19 | 1.23 | 0.94 | 0.31 | 0.99 | 1.34 | 1.21 | 1.23 | 1.17 | 0.39 | 1.05 | 1.33 | 1.19 | 0.43 | 1.12 | 1.54 | 0.99 | 0.35 | 0.94 | 1.06 | 0.89 | 1.11 | 0.56 | 1.44 | 0.97 | 1.03 | 0.90 | 1.10 | 0.97 | 1.03 | 0.91 | 1.09 | 0.98 | 1.02 | 0.52 | 0.97 | 0.72 | 1.15 | 1.39 | 1.25 | 0.70 | 1.28 | 1.08 | 0.94 |
| Galeopterus variegatus(Sunda flying lemur) | 0.97 | 1.03 | 0.50 | 0.81 | 0.84 | 1.12 | 0.45 | 2.27 | 1.12 | 1.34 | 0.53 | 0.76 | 0.93 | 0.51 | 1.79 | 1.17 | 1.23 | 0.96 | 0.29 | 0.98 | 1.37 | 1.21 | 1.24 | 1.18 | 0.37 | 1.06 | 1.34 | 1.20 | 0.41 | 1.14 | 1.54 | 0.99 | 0.33 | 0.94 | 1.06 | 0.88 | 1.12 | 0.54 | 1.46 | 0.98 | 1.02 | 0.90 | 1.10 | 0.97 | 1.03 | 0.88 | 1.12 | 0.97 | 1.03 | 0.51 | 1.00 | 0.68 | 1.18 | 1.35 | 1.28 | 0.70 | 1.26 | 1.05 | 0.99 |
| Loxodonta africana(African savanna elephant) | 0.99 | 1.01 | 0.55 | 0.84 | 0.88 | 1.10 | 0.47 | 2.15 | 1.14 | 1.30 | 0.56 | 0.80 | 0.93 | 0.55 | 1.72 | 1.17 | 1.21 | 0.98 | 0.30 | 0.99 | 1.34 | 1.23 | 1.20 | 1.18 | 0.39 | 1.07 | 1.31 | 1.19 | 0.43 | 1.14 | 1.51 | 0.99 | 0.36 | 0.93 | 1.07 | 0.88 | 1.12 | 0.55 | 1.45 | 0.97 | 1.03 | 0.93 | 1.07 | 0.98 | 1.02 | 0.92 | 1.08 | 1.02 | 0.98 | 0.52 | 0.91 | 0.69 | 1.12 | 1.44 | 1.31 | 0.70 | 1.23 | 1.08 | 0.98 |
| Orycteropus afer afer(Aardvark) | 0.95 | 1.05 | 0.50 | 0.83 | 0.84 | 1.12 | 0.48 | 2.23 | 1.12 | 1.35 | 0.52 | 0.75 | 0.94 | 0.52 | 1.78 | 1.16 | 1.24 | 0.95 | 0.30 | 0.99 | 1.36 | 1.21 | 1.23 | 1.17 | 0.40 | 1.06 | 1.34 | 1.19 | 0.41 | 1.13 | 1.54 | 0.97 | 0.37 | 0.93 | 1.07 | 0.88 | 1.12 | 0.56 | 1.44 | 0.97 | 1.03 | 0.89 | 1.11 | 0.97 | 1.03 | 0.89 | 1.11 | 0.98 | 1.02 | 0.54 | 1.04 | 0.71 | 1.20 | 1.30 | 1.22 | 0.70 | 1.30 | 1.03 | 0.96 |

Most preferred codons in the EBOV and the potential hosts are underlined; Preferentially employed codons (RSCU > 1.00) in EBOV are marked in bold; AU rich preferentially employed codons in EBOV are marked in italic. The common names of the potential hosts are provided in parenthesis. Hosts marked in red indicate African mammals.
